# Does workplace telepressure get under the skin? Protocol for an ambulatory assessment study on wellbeing and health-related physiological, experiential, and behavioral concomitants of workplace telepressure

**DOI:** 10.1186/s40359-023-01123-4

**Published:** 2023-05-03

**Authors:** Raphaël Semaan, Urs M. Nater, Raphaël Heinzer, José Haba-Rubio, Peter Vlerick, Ruben Cambier, Patrick Gomez

**Affiliations:** 1 Center for Primary Care and Public Health (Unisanté), Department of Occupational and Environmental Health, University of Lausanne, Lausanne, Switzerland; 2 Department of Clinical and Health Psychology, University of Vienna, Vienna, Austria; 3 University Research Platform “The Stress of Life – Processes and Mechanisms Underlying Everyday Life Stress”, University of Vienna, Vienna, Austria; 4 Center for Investigation and Research on Sleep, Department of Medecine, Lausanne University Hospital (CHUV) and University of Lausanne, Lausanne, Switzerland; 5 Department of Work, Organisation and Society, Ghent University, Ghent, Belgium

**Keywords:** Ambulatory assessment, Heart rate variability, Information and communication technology, Perseverative cognition, Salivary cortisol, Salivary alpha-amylase, Salivary dehydroepiandrosterone, Sleep, Workload, Workplace telepressure

## Abstract

**Background:**

The daily working life of many employees requires the use of modern information and communication technology (ICT) devices such as computers, tablets, and smartphones. The double-edged nature of digital work environments has been increasingly highlighted. Benefits such as increased flexibility come at a personal cost. One of the potential downsides is workplace telepressure, i.e., the experience of urge and preoccupation to quickly reply to work-related messages and demands using ICT. There is initial − mainly survey-based−evidence that workplace telepressure may have negative effects on a variety of wellbeing and health outcomes.

**Aims and hypotheses:**

Adopting the Effort-Recovery Model and the concept of allostatic load as theoretical frameworks, the present study aims to investigate the hypothesis that workplace telepressure is significantly associated with increased “wear and tear”, in the form of more psychosomatic complaints, worse sleep quality (self-reported and actigraphy-based), worse mood, and biological alterations (lower cardiac vagal tone, lower anabolic balance defined as the ratio of salivary dehydroepiandrosterone to salivary cortisol, and higher salivary alpha-amylase). Additionally, the study aims to investigate the hypothesis that connection to work defined as work-related workload and work-related perseverative cognition plays a significant role in the mediation of these relationships.

**Methods:**

To test our hypotheses, we will conduct an ambulatory assessment study with a convenience sample of 120 healthy workers regularly using ICTs for job communication. For one week, participants will be asked to complete electronic diaries assessing their level of workplace telepressure, psychosomatic complaints, sleep quality, mood, work-related workload, and work-related perseverative cognition. They will also continuously wear the Bittium Faros 180L ECG monitor, the wrist-worn actigraph MotionWatch 8, and perform saliva sampling five times per day.

**Discussion:**

This study will be the most comprehensive ambulatory investigation of workplace telepressure and its psychophysiological concomitants to date and constitutes an important step towards understanding how high levels of workplace telepressure may lead in the long term to secondary alterations (e.g., hypertension, chronic inflammation) and disease (e.g., heart disease). The findings of this study are also anticipated to contribute to guiding the development and implementation of interventions, programs, and policies relevant to employees’ digital wellbeing.

**Supplementary Information:**

The online version contains supplementary material available at 10.1186/s40359-023-01123-4.

## Background

### Modern information and communication technologies at work

Modern information and communication technology (ICT) devices such as computers, laptops, tablets, and smartphones are important tools in the daily working life of many employees [1, 2]. ICT has been transforming the way many people work by creating the conditions for work to take place anywhere at any time [3]. In particular, smartphones serve as small computers that include numerous functions such as digital calendars, phone calls, internet and social media access, and especially sending and receiving emails and text messages [4, 5].

ICT-mediated communication and especially email communication is essential in many organizations [4]. Message-based ICTs such as email and text messages may increase flexibility and convenience in responding to work-related requests and facilitate team collaboration across geographic and other accessibility barriers [6, 7]. With the Covid-19 outbreak, a sudden shift from office-centric workplaces to remote work took place [8, 9], a phenomenon labeled “forced flexibility” by Franken and colleagues [10]. With this shift to working from home, the number of work emails sent during both work and non-work hours has dramatically increased [11]. Emails and voice mails, as asynchronous forms of communication, allow the receiver flexibility and control in choosing when and where to handle received messages. However, as shown by several surveys and studies [12–17], many employees have limited or no response flexibility and feel the need to be continuously connected to the workplace and to respond promptly to work-related communication through ICT during work hours and off-job time − a phenomenon called the autonomy paradox [18]. For example, a qualitative study reported that insurance company employees were required to respond to customer chat messages within 15 s [14], and a survey found that 38% of Australian workers checked their emails during non-work hours and kept their mobile phones switched on [13]. To better characterize the ambivalence of employees’ relationship to ICTs and mobile technology in general, Vanden Abeele [19] recently introduced the concept of digital wellbeing. According to her model, digital wellbeing refers to “a subjective individual experience of optimal balance between the benefits and drawbacks obtained from mobile connectivity” (p. 938). One possible challenge in achieving and maintaining digital wellbeing is workplace telepressure (WTP).

### The concept of workplace telepressure

Barber and Santuzzi [20] introduced the concept of WTP to describe the preoccupation with and urge for responding quickly to work-related ICT messages. As such, WTP is a psychological state experienced by the employee. Both personal factors such as neuroticism and workaholism and organizational factors such as prescriptive norms appear to contribute to the experience of WTP [14, 20–23]. Workplace telepressure is supposed to emerge when workers begin to view the use of asynchronous communication technologies as similar to synchronous communication forms (e.g., face-to-face communication), which generally require immediate responses. As the employees prioritize ICT-assisted communications during worktime and off-job time, the response flexibility and control over response times that asynchronous communication would normally allow are canceled out. Work periods without interruptions that would be required to accomplish work tasks as well as necessary uninterrupted time for recovery become less frequent and shorter. Workplace telepressure can ultimately lead employees to perceive the use of message-based technology for work purposes as inescapable work instead of flexible work access [20]. Since the initial work by Barber and Santuzzi, researchers have shown a growing interest in studying WTP (e.g., [22, 24–36]).

### Workplace telepressure, connection to work, and wellbeing and health

**Fig. 1:**
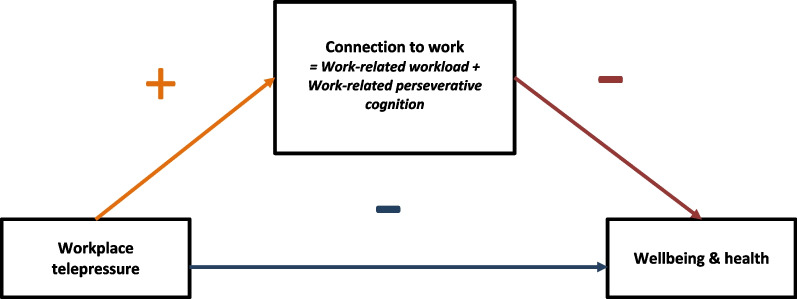
Conceptual model linking workplace telepressure to wellbeing and health through connection to work. The minus sign indicates a negative relationship. The plus sign indicates a positive relationship

There is growing evidence that high levels of WTP might represent a significant risk factor for employees’ wellbeing and health. For instance, employees reporting higher levels of WTP also reported higher levels of burnout, absenteeism due to physical or mental health issues, and worse satisfaction with work-life balance (e.g., [20, 23, 24, 37]). In the proposed project, we aim to further examine the potential effects of WTP on wellbeing and health by investigating how WTP is related to important indices of wellbeing and health that have been only partially or not yet considered in research on WTP. Drawing from the Effort-Recovery Model [38], we suggest that WTP can deteriorate employees’ wellbeing and health by prolonging employees’ work-related psychophysiological effort expenditure and by impairing psychophysiological recovery. The Effort-Recovery Model posits that work-related demands require effort, which strains employees’ psychophysiological systems. During non-work time, the psychophysiological systems can revert to pre-demand states as long as employees refrain from putting additional strain on their psychophysiological systems. If employees’ psychophysiological systems recover sufficiently, there should be no long-term negative consequences for employees’ wellbeing and health. In contrast, if exposure to work-related demands is prolonged, recovery is likely to be insufficient. This imbalance is expected to result in an accumulation of psychophysiological alterations − also known as "wear and tear" within the concept of allostatic load [39] − that can deteriorate employees’ wellbeing and health. We hypothesize that compared to lower levels of WTP, higher levels of WTP are associated with a more unfavorable wellbeing and health profile. Moreover, we aim to uncover potential underlying mechanisms of the hypothesized relationships between WTP and the wellbeing and health-related measures. Barber and Santuzzi [20] suggested that WTP might be a critical factor for employees’ wellbeing and health because it has the potential to extend employees’ work stress both during designated work times and during non-work times by encouraging continued connection to work activities. We hypothesize that the preoccupation and urge to respond to message-based ICTs for work purposes that defines WTP prolong employees’ work-related psychophysiological effort expenditure and impair psychophysiological recovery by increasing connection to work. We operationalize connection to work in terms of work-related workload and work-related perseverative cognition. Our conceptual model is depicted in Fig. 1.

To address these questions, we plan to use ambulatory assessment methods, which in contrast to the more common cross-sectional survey studies allow for the measurement of human behavior where and when it happens and for the analysis of day-level within-person associations [40, 41]. Below, we introduce the wellbeing and health-related outcomes and connection to work with its two components “work-related workload” and “work-related perseverative cognition” as potential mediating factors.

In reviewing research on WTP, we use “workplace telepressure” when referring to this concept in a broad sense, “general workplace telepressure” to describe the WTP that employees report to experience in general in their life, and “daily workplace telepressure” to describe the WTP that employees report to experience during a specific day. Most researchers have considered only general WTP. Cambier and colleagues [42, 43] showed that there is substantial within-person variability in daily WTP (around 50%); this finding points to the importance of assessing daily WTP and thus the need for ambulatory assessment studies.

### Wellbeing and health-related outcomes

#### Biological parameters

The hypothalamic–pituitary–adrenal (HPA) axis is a central regulatory system implicated in the organism’s reaction to stressors [44–46]. Cortisol and dehydroepiandrosterone (DHEA) are the main products of the HPA axis [47]. Cortisol and DHEA exhibit the highest levels after awakening, followed by a decline throughout the afternoon and evening [48]. Psychosocial stressors elicit in most healthy people an activation of the HPA axis resulting in increased salivary cortisol (sC) and salivary DHEA (sDHEA) secretion [49–54]. Dysregulation of the HPA axis in the form of abnormal cortisol and/or DHEA responses to stressors has been linked to several health problems [55–57]. Anabolic balance is the ratio of DHEA to cortisol and has been suggested to be a sensitive indicator of wellbeing and health, more so than assessing only cortisol or DHEA [52, 58, 59]. Lower anabolic balance is associated with more unfavorable wellbeing and health outcomes [58, 60].

A second main regulatory system involved in the response to stressors is the sympathoadrenal-medullary (SAM) axis [44, 61]. Salivary alpha-amylase (sAA) is an enzyme secreted from the salivary glands that has gained interest over the last fifteen years as a marker of the SAM axis activity [62–64]. SAA activity is low in the morning and steadily increases over the course of the day, typically reaching its peak in the late afternoon [65]. In healthy individuals, psychosocial stressors induce increased sAA activity (e.g., [50, 64, 65]).

Heart rate variability (HRV) represents the change in the time interval between successive heartbeats. Its assessment is of particular interest because HRV can provide an index of the activity of the parasympathetic nervous system [66], which is associated with many psychophysiological processes [18, 67, 68]. Low cardiac parasympathetic activity is an important predictor of disease and mortality [69–72]. We refer to parasympathetic activity of the heart as cardiac vagal tone [73].

The four parameters sC, sDHEA, sAA, and HRV index the activity of three intertwined yet distinct biological stress-related systems. Together, these systems provide a comprehensive and complementary in-depth picture of the biological response to short-term changes in psychosocial stress factors and are thus well-suited for investigating the effects of day-to-day variations in WTP. Although there is reasonable theoretical justification for an association between WTP and HPA axis, SAM axis, and cardiac parasympathetic activity, there is no empirical evidence yet.

#### Psychosomatic complaints

Psychosomatic complaints refer to self-reported health problems such as musculoskeletal pain and headache [74]. Psychosomatic complaints are very common in the general population [75–77] and are frequent reasons reported for health care utilization and for sick leave [75, 77, 78]. A few findings indirectly suggest that WTP might be significantly associated with psychosomatic complaints [20, 35, 79].

In this study we integrate cognitive weariness, a core component of burnout assessment, into the concept of psychosomatic complaints. Cognitive weariness is defined as the difficulty to maintain and optimize cognitive and intellectual abilities over time on sustained cognitive demands [80]. General WTP has been associated with increased cognitive weariness [20, 35, 79]. The planned study is the first one to investigate the relationship between WTP and psychosomatic complaints using an ambulatory assessment approach.

#### Sleep quality

Researchers in occupational health psychology have been increasingly acknowledging the importance of studying the associations among the three major areas of life: work, non-work, and sleep [7]. Sleep disturbances adversely affect physical and mental health [81–85].

Compared to the gold standard of polysomnography, actigraphy is considered a good low-cost, non-invasive, objective approach to continuously monitoring sleep behavior [86]. Actigraphy-derived sleep fragmentation at night has been shown to be sensitive to work stressors [87, 88]. Subjective sleep measures and actigraphy-based sleep parameters are not highly correlated [89–91].

Three cross-sectional survey studies found that higher levels of general WTP were significantly correlated with poorer self-reported sleep quality [20, 35, 79]. No ambulatory data exist on the association between WTP and both self-reported sleep quality and actigraphy-based sleep parameters.

#### Mood

Moods are important components of subjective wellbeing [92–94]. Park and colleagues [95] reported that general WTP predicted higher levels of negative affect across a five-week period but did not report statistical analyses. No ambulatory data exist on the association between WTP and mood.

### The mediating factor: connection to work

Drawing from Barber and Santuzzi [20], we hypothesize that WTP might impair employees’ wellbeing and health by encouraging continued connection to work during designated work times and during non-work times. Some support for this contention comes from the psychological detachment literature. Psychological detachment from work refers to “the individual’s sense of being away from the work situation” ([96] p. 579). Increased connection to work means that psychological detachment from work is impaired. Higher levels of general WTP are associated with less general psychological detachment from work [20, 23, 35, 79]. Moreover, Santuzzi and Barber [35] found that general WTP was indirectly related to burnout and poorer sleep quality through psychological detachment at the between-person level. In a five-day diary study, Cambier and colleagues [42] found that the negative association between WTP during off-job hours and psychological detachment during off-job hours was significant at the between-subject level but not at the within-subject level. Lack of psychological detachment results from performing work activities, from not disconnecting mentally from work during breaks and before and after work, or from a combination of the two [97–99]. In this project, we aim to extend the existing literature on WTP as we consider the possible association between WTP and connection to work by operationalizing connection to work in terms of work-related workload and work-related perseverative cognition.

#### Work-related workload

Urges are difficult to resist [100]. Consequently, employees might be expected to give in to their urges and thus engage more frequently in behaviors such as checking, reading, and writing emails and text messages when experiencing high levels of WTP than when experiencing low levels of WTP. Work-related electronic communication may often entail requests that generate additional work in the form of calls or other tasks such as web-browsing for work-related purposes and using computer software to perform tasks such as text processing. Thus, we would predict that higher levels of WTP are associated with more time spent on work activities. In line with these ideas, survey studies have shown that employees who reported higher levels of general WTP also reported to respond more frequently to work emails during both work and non-work hours, vacation days, and even sick days than employees with lower levels of general WTP [20, 23, 37]. Furthermore, employees with higher levels of general WTP exhibited shorter response latencies to work emails during work hours [20, 23]. In a diary study, Van Laethem and colleagues [101] found that employees displaying higher levels of general WTP reported significantly more work-related smartphone use both during work and after work than employees with lower levels of general WTP. In another diary study, Cambier and colleagues [42] reported that daily WTP during off-job time was significantly related to daily work-related smartphone use during off-job time. Cross-sectional analyses revealed that general WTP was positively related to frequency of ICT use at work [35] and to perform work tasks and arrange work schedules at home and during non-work hours [79]. Taken together, these studies suggest that higher levels of WTP may be associated with higher work-related workload throughout a workday.

Moreover, we hypothesize that work-related workload is partially mediating the relationship between WTP and the studied measures of wellbeing and health. As suggested by the Effort-Recovery Model [38], increased demand exposure via increased working hours could exhaust employees’ resources to the point of poor wellbeing and health. Several studies have shown that long working hours adversely affect health (e.g., [102–106]). A significant linear relationship has been reported between the number of working hours and sleep disturbances [107]. Associations between longer working hours and physiological changes relevant to the planned project have been also reported. Compared to employees working regular hours, employees working long hours exhibited decreased vagal activity and increased sympathetic activity as indexed by measures of HRV [108]. Moreover, some evidence exists for incomplete or insufficient physiological recovery as a mechanism that may explain the relationship between long working hours and impaired wellbeing and health [104]. Incomplete or insufficient recovery from work, which is positively related to the number of working hours (e.g., [109]), is associated with higher cortisol levels [110] and an elevated risk of cardiovascular death [111]. With regard to ICT-assisted work tasks more specifically, ICT use for work purposes during off-job time has been associated with worse psychological wellbeing in most investigations [112], worse affect [98, 113], and poorer self-rated sleep quality [97]. Employees reporting to be contacted often or sometimes outside of regular working hours (e.g., by email) in the past 12 months exhibited a higher risk of health impairments (e.g., musculoskeletal and gastrointestinal complaints) and a higher risk of sickness absence during the same period [114]. Using a cross-sectional design, Hu and colleagues [79] found that general WTP had an indirect effect on physical exhaustion and sleep quality via the use of technology devices to perform work tasks and arrange work schedules at home and during non-work hours. The results by Hu and colleagues [79] refer to between-person associations.

#### Work-related perseverative cognition

Perseverative cognition has been defined as “repetitive or sustained activation of cognitive representations of past stressful events or feared events in the future” ([115], p. 407). Prototypical forms of perseverative cognition are future-oriented worry and past-oriented rumination [116]. Higher levels of general WTP predicted higher levels of negative rumination after work over a five-week period [95]. More recently, Cambier and Vlerick [43] embedded boundary-crossing contexts in Barber and Santuzzi’s telepressure measure, such that the new item pool assesses both WTP during leisure time and private life telepressure (PTP, i.e., the preoccupation with and urge for responding quickly to personal ICT messages) at work. Their findings revealed a positive association between WTP during leisure time and work-related rumination during leisure time, that is, employees tended to ruminate more about work-related issues during leisure time on days when they experienced more WTP during leisure time. These authors found a similar association between PTP at work and private life rumination at work. Perseverative cognition has been identified as a common cognitive process likely to play a significant role in both psychological and somatic health [116]. There is evidence that perseverative cognition is associated with higher cortisol levels, higher sAA activity, and lower cardiac vagal tone [50, 116, 117]. The link between perseverative cognition and DHEA remains to be investigated. Perseverative cognition is positively and prospectively associated with the number of subjective health complaints [115, 118–121], worse sleep quality [101, 122, 123], and worse mood [116, 121, 124, 125].

### Aims and hypotheses

**Fig. 2:**
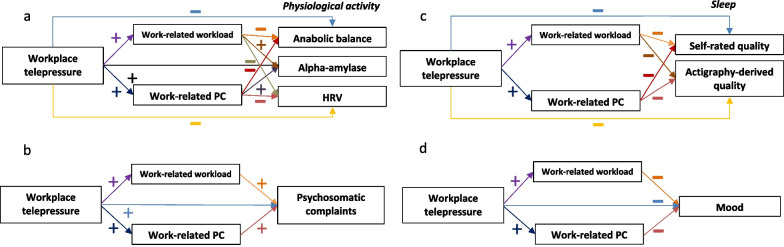
Proposed model showing work-related workload and work-related perseverative cognition as linking mechanisms between workplace telepressure and **a** physiological activity, **b** psychosomatic complaints, **c** sleep quality, and **d** mood. The minus sign indicates a negative relationship. The plus sign indicates a positive relationship. *PC* perseverative cognition, *HRV* heart rate variability

This project aims to contribute to further our knowledge on the timely topic of WTP by testing a model that proposes that WTP is significantly associated with a more unfavorable profile of wellbeing and health-related measures, and that these associations are mediated by connection to work. We have the following four hypotheses (Fig. 2).

#### Hypothesis 1

1.1 Higher levels of WTP are associated with lower anabolic balance, higher sAA activity, and lower HRV, and 1.2 work-related workload and work-related perseverative cognition are significant mediators of these relationships (Fig. 2a).

#### Hypothesis 2

2.1 Higher levels of WTP are associated with more psychosomatic complaints, and 2.2 work-related workload and work-related perseverative cognition are significant mediators of this relationship (Fig. 2b).

#### Hypothesis 3

3.1 Higher levels of WTP are associated with both worse self-rated and actigraphy-derived sleep quality, and 3.2 work-related workload and work-related perseverative cognition are significant mediators of these relationships (Fig. 2c).

#### Hypothesis 4

4.1 Higher levels of WTP are associated with worse mood, and 4.2 work-related workload and work-related perseverative cognition are significant mediators of this relationship (Fig. 2d).

All hypotheses are at the within-person level.

## Methods

### Participants

Participants will be employees (50% female) recruited from a variety of organizations and occupations. We will recruit the participants using flyers, the local press, and social media platforms. Based on sample size calculations (see Sect. "Sample size calculation"), we will need 120 participants with complete data to test our hypotheses. Because of potential device malfunctioning, dropouts, non-compliance of participants, or not usable data, we will schedule 10 additional participants, i.e., 130 participants in total. Participants will receive a compensation of up to 352 Swiss Francs and will be reimbursed for study-related travel expenses.

To be included in the study, prospective participants must fulfill the following criteria: (1) being healthy, (2) being over 18 years old, (3) having good French skills, (4) working a paid daytime job with a weekly regular schedule of at least four consecutive workdays within the same organization in Switzerland, and (5) using ICT daily to communicate for work-related purposes with supervisor, coworkers, subordinates, clients, or patients.

The psychophysiological variables of our study have been shown to be affected by (1) shift work [126, 127], (2) a Body Mass Index > 30 kg/m^2^ [128–130], (3) cardiovascular, neurological, metabolic, endocrine, respiratory, autoimmune, psychiatric disorders, or the sleep disorders severe insomnia and sleep apnea [127, 131–133], (4) pregnancy and breastfeeding [134–137], and (5) alcohol abuse [127, 138]. We will consider these factors as exclusion criteria. We will also exclude participants who (6) use psychotropic drugs or any medication known to affect our variables [127, 139], except hormonal contraceptives for women of childbearing age and hormonal replacement therapy drugs for postmenopausal women. We will finally exclude employees who (7) wear a pacemaker.

### Procedure

The study’s procedure for each participant will consist of three main phases: an online entry questionnaire, a laboratory visit, and an ambulatory assessment. The entire study will be conducted in French.

#### Online entry questionnaire

Participants who contact the research team will receive an email including the study information sheet and the internet link to the entry questionnaire. They will be invited to carefully read the information sheet before proceeding with the entry questionnaire. In accordance with article 9 of the Swiss Human Research Ordinance [140], participants will have to tick a box to accept that their data will be saved and treated in order to establish their eligibility for this study.

#### Laboratory visit

Eligible participants will be invited to our laboratory for an initial meeting. After being explained about all the procedures to be undertaken and signing the consent form, they will be asked to fill out baseline questionnaires. These questionnaires will allow us to better characterize the sample and to control statistically for potential confounding variables. Afterwards, participants will be familiarized with the questionnaires, instruments, and procedure of the ambulatory assessment. At the end of the meeting, they will leave with a briefcase containing the material for the ambulatory assessment.

#### Ambulatory assessment

Participants will be monitored during seven days. The assessment will be scheduled during a workweek that is expected to be typical for each employee. The participation week will be scheduled so that the weekend days are assessed consecutively. Furthermore, the participation week will not be preceded or followed by vacations given the effects that these can have on employees [141]. For women of childbearing age, the ambulatory assessment phase will start following the end of their period. Data collection will end with the morning questionnaires of the eighth day to assess sleep-related variables of the night before.

Participants will be able to contact the research staff throughout the assessment period should the need arise. At the end of the assessment period, an investigator will meet the participants at a mutually agreed upon location to pick up the material.

### Measures

The lists of all measures with detailed information are given in the Supplementary Material (Additional file 1).

#### Online entry questionnaire measures

##### Sociodemographic data

Ad-hoc questions will be used to assess the following sociodemographic data: age, sex, body height, body weight, mother tongue, French skills, workweek schedule, average number of actual work hours per week, frequency of ICT use for work-related communication during the workweek and the weekend, work on weekends according to contract, on-call hours according to contract, and shift work.

##### Health-related data

We will ask participants to report any known current disease or medical condition. Additionally, we will ask specifically for sleep apnea with one question and assess insomnia using the 7-item Insomnia Severity Index [142, 143]. One sample item reads as follows, “To what extent do you consider your sleep problem to interfere with your daily functioning (e.g., daytime fatigue, ability to function at work/daily chores, concentration, memory, and mood)?” (Cronbach’s α = 0.74; [142]) and is scored on a 5-point Likert scale (0 = “Not at all interfering” to 4 = “Very much interfering”). The total score ranges from 0 to 28, with higher scores indicating more severe insomnia: 0–7 = No clinically significant insomnia, 8–14 = Subthreshold insomnia, 15–21 = Clinical insomnia, and 22–28 = Severe insomnia). We will also ask participants to list any medication intake and to indicate if they smoke (e.g., cigarettes, e-cigarettes, pipes, smokeless tobacco), take recreational/psychotropic drugs (e.g., stimulant drugs, opioids, anabolic steroids), wear a pacemaker, are pregnant, or are lactating. Finally, we will assess alcohol abuse/dependence during the past six months using the 6-item Alcohol Abuse/Dependence Module of the Patient Health Questionnaire [144, 145].

##### General workplace telepressure and private life telepressure measures

Although the concept of telepressure has been first developed in the context of message-based technology use for work purposes, people can experience telepressure also when using message-based technology for non-work purposes. We will measure general WTP and general PTP using adapted versions of the 6-item WTP measure [20]. The adaptation consists in adding “work-related” and “personal”, respectively, in five of the six items. One sample item reads as follows, “I can’t stop thinking about a [work-related] / [personal] message until I’ve responded”. All items are scored on a 5-point Likert scale (1 = “Strongly disagree” to 5 = “Strongly agree”). The mean score ranges from 1 to 5, with higher scores indicating higher levels of general WTP and PTP. Cronbach’s alphas of our adapted general WTP and PTP measures were 0.89 and 0.90, respectively, in a sample of 75 employees.

#### Laboratory visit measures

##### Complementary sociodemographic and health-related data

Additional sociodemographic data will include marital status, total number of individuals living in the household, total number of adults living in the household, total number of children under 18 years old currently living in the household, educational level, current occupation/job, managerial/supervisory role, place of work, and job tenure. We will ask women of childbearing age to indicate the average length of their menstrual cycle, their average period length, and the first day of their last period. This information will be used to ascertain that their ambulatory assessment phase is scheduled outside of their period.

##### Workplace fear of missing out

Workplace fear of missing out will be assessed using the 10-item workplace Fear of Missing Out scale [37]. One sample item reads as follows, “I worry that I will not know what is happening at work” (α = 0.90–0.94; [33, 37]) and is preceded by the stem “When I am absent or disconnected from work…”. All items are scored on a 5-point Likert scale (1 = “Strongly disagree” to 5 = “Strongly agree”). The mean score ranges from 1 to 5, with higher scores indicating stronger workplace fear of missing out.

##### Psychological detachment from work

We will use the 4-item psychological detachment subscale of the recovery experience questionnaire [146] to assess psychological detachment from work. One sample item reads as follows, “I don’t think about work at all” (α = 0.84–0.89; [32, 146]) and is preceded by the stem “During time after work:”. All items are scored on a 5-point Likert scale (1 = “I do not agree at all” to 5 = “I fully agree”). The mean score ranges from 1 to 5, with higher scores indicating better psychological detachment.

##### ICT-related response expectations and availability

*Response expectations.* ICT-related response expectations of employees will be assessed with the 2-item response expectations subscale of the ICT Demand Scale [147]. One sample item reads as follows, “I am expected to respond to e-mail messages immediately” (α = 0.78–0.86; [20, 23, 147]). Items are scored on a 5-point Likert scale (0 = “Never” to 4 = “Almost always”). Mean scores range from 0 to 4, with higher scores indicating higher response expectations.

*Availability.* ICT-related availability of employees will be assessed with the 4-item availability subscale of the ICT Demand Scale [147]. One sample item reads as follows, “I’m contacted about work-related issues outside of regular work hours” (α = 0.71–0.83; [20, 23, 147]). Items are scored on a 5-point Likert scale (0 = “Never” to 4 = “Almost always”). Mean scores range from 0 to 4, with higher scores indicating higher availability.

##### Technostress creators

Technostress creators are factors that induce stress due to the use of ICTs. According to Ragu-Nathan and colleagues [148], they can be grouped into five technostress dimensions, i.e., techno-overload, techno-invasion, techno-complexity, techno-insecurity, and techno-uncertainty. We will assess these five dimensions with the 21-item technostress creators scale [148, 149]. One sample item of techno-overload reads as follows, “I am forced by this technology to work much faster” (α = 0.90). One sample item of techno-invasion reads as follows, “I feel my personal life is being invaded by this technology” (α = 0.88). One sample item of techno-complexity reads as follows, “I do not know enough about this technology to handle my job satisfactorily” (α = 0.88). One sample item of techno-insecurity reads as follows, “I have to constantly update my skills to avoid being replaced” (α = 0.84). One sample item of techno-uncertainty reads as follows, “There are constant changes in computer software in our organization” (α = 0.91). All items are scored on a 7-point Likert scale (1 = “Strongly disagree” to 7 = “Strongly agree”). For all dimensions, mean scores range from 1 to 7, with higher scores indicating more technostress creators.

##### Workaholism

We will assess workaholism using the two working excessively and working compulsively subscales of the 10-item short version of the Dutch Work Addiction Scale [150, 151]. One sample item of the working excessively subscale reads as follows, “I find myself continuing work after my co-workers have called it quits” (α = 0.65–0.81; [20, 150, 151]). One sample item of the working compulsively subscale reads as follows, “I often feel that there’s something inside me that drives me to work hard” (α = 0.69–0.81; [20, 150, 151]). All items are scored on a 4-point scale (1 = “(Almost) Never” to 4 = “(Almost) Always”). The mean score ranges from 1 to 4, with higher scores indicating higher levels of workaholism.

##### Segmentation preferences and supplies

Segmentation preferences refer to the degree to which employees prefer to keep aspects of work and home separated from one another [152]. We will measure this construct using the 4-item segmentation preferences scale [152]. One sample item reads as follows, “I like to be able to leave work behind when I go home” (α = 0.91, [152]).

Segmentation supplies refer to the employees’ perception of the degree to which their organization/workplace provides freedom of work-home segmentation [152]. We will assess this construct using the 4-item workplace segmentation supplies scale [152]. One sample item reads as follows, “At my workplace, people are able to prevent work issues from creeping into their home life” (α = 0.94, [152]). The items of both scales are scored on a 7-point Likert scale (1 = “Strongly disagree” to 7 = “Strongly agree”). The mean score ranges from 1 to 7, with higher scores indicating stronger segmentation preferences and norms in the organization.

##### Personality traits

The two personality traits neuroticism and conscientiousness will be assessed using respectively eight and nine items of the Big Five Inventory [153–155]. One sample item assessing neuroticism reads as follows, “Gets nervous easily” (α = 0.82, [155]). One sample item assessing conscientiousness reads as follows, “Perseveres until the task is finished” (α = 0.80, [155]). The items are preceded by the stem, “I see myself as someone who…”. All items are scored on a 5-point Likert scale (1 = “Disagree strongly” to 5 = “Agree strongly”). The mean score for each scale ranges from 1 to 5, with higher scores indicating more neuroticism and conscientiousness.

##### Depression, anxiety, and stress

We will assess depressive symptoms, anxiety, and stress with the 21-item Depression, Anxiety, and Stress Scale [156]. One sample item assessing depression reads as follows, “I felt I wasn’t worth much as a person” (α = 0.91, [156]). One sample item assessing anxiety reads as follows, “I felt I was close to panic” (α = 0.84, [156]). One sample item assessing stress reads as follows, “I found it difficult to relax” (α = 0.90, [156]). All items are scored on a scale from 0 = “did not apply to me at all” to 3 = “applied to me very much or most of the time”. The total score of each subscale ranges from 0 to 21, with higher scores indicating worse depression, anxiety, and stress outcomes over the past week.

##### Trait mindfulness

Attention and awareness in daily life will be assessed using the 15-item Mindful Attention Awareness Scale [157, 158]. This questionnaire requires the respondent to evaluate the frequency of different everyday life experiences using a 6-point Likert scale (1 = “Almost never” to 6 = “Almost always”). One sample item reads as follows, “I rush through activities without being really attentive to them” (α = 0.84, [158]). The mean score ranges from 1 to 6, with higher scores indicating higher levels of dispositional mindfulness.

Finally, we will ask participants to fill out the general WTP measure and the general PTP measure again in order to evaluate the test–retest reliability of these two instruments.

#### Ambulatory assessment measures

The daily schedule of the ambulatory assessment measures is shown in Table 1. It consists of five sampling occasions during wake time: (1) immediately after awakening while still lying in bed, (2) 30 min after awakening, (3) 12:30 p.m. (± 30 min), (4) 5:30 p.m. (± 30 min), and (5) bedtime. Participants will be asked to complete questionnaires four times per day (30 min after awakening, 12:30 p.m., 5:30 p.m., and bedtime) with an iPad Mini 2 (Apple Inc.) using the software iDialogPad developed by G. Mutz at the University of Cologne. The full iDialogPad script is given in the Supplementary Material. The iPad’s screen will have a blue light filter to ensure that the evening surveys do not expose participants to the artificial light that can affect their sleep. Sampling times will be automatically registered on the iPad.

**Table 1:** Assessment of daily self-reported measures (number of items in parentheses), physiological measures, and actigraphy during a participation day

Daily Measures	AW	AW + 30 min	12:30 p.m	5:30 p.m	Bedtime	Sleep
WTP^1^ (6)			X	X	X	
PTP^2^ (6)			X	X	X	
Work-related workload^1^ (5)			X	X	X	
Private life workload^2^ (3)			X	X	X	
Work-related PC^1^ (4)			X	X	X	
Private life PC^2^ (4)			X	X	X	
Number of stressful events^2^ (1)			X	X	X	
Qualitative^1^ and quantitative^2^ aspects of sleep (11)		X				
Biobehavioral measures^2^ (6)		X	X	X	X	
Psychosomatic complaints^1^ (12)		X	X	X	X	
Mood^1^ (8)		X	X	X	X	
Saliva sampling^1^	X	X	X	X	X	
ECG^1^ (Bittium Faros 180L)	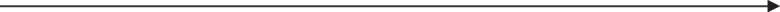					
Actigraphy^1^ (MotionWatch 8)	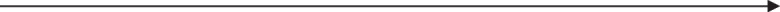					

*AW* awakening, *PC* perseverative cognition, *PTP* private life telepressure, *WTP* workplace telepressure

^1^Variables of interest

^2^Control variables. → Refers to continuous recording during wake time and sleep time

Participants will collect their saliva on each sampling occasion for sC, sDHEA, and sAA assessment. They will also continuously wear the MotionWatch 8 (CamNtech Ltd., Cambridgeshire, England) and Bittium Faros 180L ECG monitor (Bittium Corporation, Oulu, Finland) for actigraphic and electrocardiographic recordings, respectively.

##### Daily workplace telepressure and private life telepressure measures

We will assess daily WTP and daily PTP with the 6-item WTP and the 6-item PTP measures adjusted for repeated measurement during a day, respectively. The verb tense of the items is changed from present tense to past tense and a time reference is given (“Since the last assessment…”). One sample item reads as follows, “It was difficult for me to resist responding to a work-related message right away”. The scoring of these measures is the same as the scoring of the general WTP and general PTP measures.

##### Work-related workload

We will assess work-related workload using the 3-item workload measure of Derks and colleagues [159]. One sample item reads as follows, “I had to work extra hard to finish things” (α = 0.91, [159]). We adapted the instruction to ensure that only work-related workload is assessed. The instruction reads as follows, “Please rate how much you agree or disagree with the following statements. Refer only to work-related activities that you have performed since the last assessment.” All items are scored on a 5-point Likert scale (1 = “Totally disagree” to 5 = “Totally agree”). The mean score ranges from 1 to 5, with higher scores indicating higher work-related workload. Additionally, we will ask participants to report (1) the total time spent performing work activities and (2) the time (in percentage) spent working at the workplace, at home, or other places since the last assessment (e.g., 50% at the workplace, 40% at home, and 10% in other places).

##### Private life workload

We adapted the 3-item work-related workload measure of Derks and colleagues [159] to assess private life workload. The three items remain the same as in Derks and colleagues [159] but the instruction is altered to ensure that only private life workload is assessed. The instruction reads as follows, “Please rate how much you agree or disagree with the following statements. Refer only to activities at home/in your private life that you have performed since the last assessment.” The scoring of the scale is the same as the scoring of the work-related workload measure. Higher scores indicate higher private life workload.

##### Work-related perseverative cognition

We will use the 3-item work-related worry/rumination measure [160] to assess work-related perseverative cognition. One sample item reads as follows, “My thoughts kept returning to a stressful situation at work” (α = 0.74, [160]). Participants will be asked to rate the extent to which they experienced such thoughts since the last assessment. All items are scored on a 5-point Likert scale (1 = “Not at all” to 5 = “A great deal”). The mean score ranges from 1 to 5, with higher scores indicating more work-related worry and rumination. Additionally, participants will estimate the total duration of their work-related worry/rumination since the last assessment.

##### Private life perseverative cognition

We adapted the 3-item work-related worry/rumination measure of Flaxman and colleagues [160] to assess private life perseverative cognition. One adapted item reads as follows, “My thoughts kept returning to a stressful situation in my private life*”*. The scoring remains the same as in the original scale. Additionally, participants will estimate the total duration of their private life perseverative cognition since the last assessment.

##### Number of stressful events

Participants will be asked to report the number of stressful events they experienced since the last assessment. They will be provided with the following definition of stressful events: “Stressful events are minor and major events that have made you feel tense, irritated, angry, sad, disappointed, or negative in any other way” [121].

##### Sleep

We will use an 11-item sleep questionnaire to assess different daily aspects of sleep. *Qualitative aspects of sleep* will be measured with five items inspired by the Karolinska Sleep Index [161, 162], the Spiegel Sleep Questionnaire [163], and the St. Mary’s Hospital Sleep Questionnaire [164]. The five items cover central qualitative aspects of sleep (overall sleep quality, restless sleep, difficulty falling asleep, difficulty maintaining sleep, and premature awakening). All items are scored on a 5-point Likert scale. The total score ranges from 5 to 25, with higher scores indicating better subjective sleep quality. *Quantitative aspects of sleep* will be measured with five items from the St. Mary’s Hospital Sleep Questionnaire. Quantitative aspects of sleep include bedtime, sleep onset latency, waking time, getting-out-of-bed time, and sleep duration. Additionally, the mode of awakening will be assessed using the following question, “This morning, did you wake up spontaneously/naturally?”. Response options are “Yes” and “No”.

##### Biobehavioral measures

Following previous methods [50, 165, 166], we will ask the participants to report the number of caffeinated beverages, alcoholic beverages, tobacco products, e-cigarettes as well as any drugs and medication consumed since the last assessment.

##### Psychosomatic complaints

The questionnaire assessing psychosomatic complaints consists of two subscales: one assessing somatic complaints and the other cognitive weariness. *Somatic complaints* will be measured with seven items from the Somatic Symptom Scale-8 [167]. One sample item reads as follows, “Back pain”. *Cognitive weariness* will be assessed using the 5-item cognitive weariness subscale of the Shirom-Melamed Burnout Measure [80]. One sample item reads as follows, “I have difficulty concentrating” (α = 0.93, [80]). All items are preceded by the following question: “At this moment, how much are you bothered by any of the following problems?” and scored on a 5-point Likert scale (0 = “Not at all” to 4 = “Very much”). The total score ranges from 0 to 48, with higher scores indicating more severe psychosomatic complaints.

##### Mood

Following the conceptualization of Matthews and colleagues [168] and Schimmack and Grob [169], the three basic dimensions of mood, valence, calmness, and energetic arousal will be measured using an 8-item mood scale, which is a modified version of the 6-item mood scale developed by Wilhelm and Schoebi [170]. Two items have been added to the original 6-item scale following recommendations of P. Wilhelm (personal communication, 17.10.2022). The initial instruction reads as follows, “At this moment I am/feel:”. One sample item of the 3-item valence subscale is “1. Unwell - 8. Well”, one sample item of the 3-item calmness subscale is “1. Agitated - 8. Calm”, and one sample item of the 2-item energetic arousal subscale is “1. Full of energy - 8. Without energy”. All items are scored on an eight-point bipolar scale (1 = “Extremely” to 4 = “Rather”; 5 = “Rather” to 8 = “Extremely”). The mean score of each dimension ranges from 1 to 8. Scores of four items are reversed in order to ensure that higher scores indicate better mood (i.e., higher positive valence, higher calmness, and higher energetic arousal).

##### Saliva sampling

In order for participants to collect their saliva five times per day (i.e., immediately after awakening while still lying in bed, 30 min after awakening, 12:30 p.m. (± 30 min), 5:30 p.m. (± 30 min), and bedtime) in a hygienic and convenient way, we will ask them to use the set of SaliCaps (IBL International, Hamburg, Germany). SaliCaps are low-bind polypropylene 2 mL cryovials that allow the collection of saliva using a polypropylene straw. Participants will be asked to follow the saliva sampling instruction displayed on the iPads. The instruction reads as follows, “First, swallow the saliva currently in your mouth. Now, hold your saliva in your mouth for two minutes. You can no longer swallow and must then transfer the accumulated saliva into the tube. Press OK to start the timer. [2 min later] Now transfer the saliva into the tube using the straw. Make sure you have sealed the tube on all sides.”. Then, they will be asked whether they were able to follow the following instructions: no drinking (other than water − at the latest 10 min before saliva sampling), eating, smoking, or engaging in vigorous physical activity in the last 30 min, and no tooth brushing in the last 60 min. The obtained saliva samples will be stored during the assessment period in a provided plastic freezer bag in the participants’ refrigerators and then kept in a freezer at − 30 °C in our laboratory before being shipped to the Biochemical Laboratory of the Department of Clinical Psychology, at the University of Vienna headed by U.M. Nater. Free sC concentrations will be measured using a Cortisol Saliva Luminescence Immunoassay (IBL-Tecan, Hamburg, Germany). DHEA concentrations will be measured using a DHEA Saliva Enzyme-Linked Immunosorbent Assay (IBL-Tecan, Hamburg, Germany). SAA activity will be measured using reagents provided by DiaSys Diagnostic Systems (Holzheim, Germany).

##### Electrocardiographic measures

The Bittium Faros 180L (Bittium Corporation, Oulu, Finland) is a lightweight (18 g), small, unobtrusive, and waterproof ECG device, equipped with a long-lasting battery allowing continuous recording for up to eight days. It is attached to the chest using three adhesive electrodes, a single ECG patch electrode, or a chest belt. The ECG will be recorded at a sampling rate of 250 Hz together with an accelerometer sampled at 25 Hz. Data will be analyzed with the Bittium Cardiac Navigator software (Bittium Corporation, Oulu, Finland) to obtain indices of HRV. The root mean square of successive differences (RMSSD) will be the main HRV index. RMSSD reflects cardiac vagal tone [72, 171] and is relatively free of respiratory influences [172].

##### Actigraphic measures

We will use the MotionWatch 8 (CamNtech Ltd., Cambridgeshire, England) to record participants’ wake and sleep periods (rest/activity cycles). The MotionWatch 8 is a lightweight (~ 10 g), small, and waterproof wristwatch-like activity-monitoring device. The watch is equipped with a light sensor and a very long-lasting battery allowing a continuous recording for up to 91 days. Participants will wear the MotionWatch 8 on the wrist of the non-dominant arm. We will also ask them to event-mark two time points that are essential for the computation of sleep quality and quantity indices: when they (i) get out of bed in the morning and (ii) are ready to sleep at night. Compliance to actigraphy event markers is generally moderate to high [173]. Movement of the wrist will be recorded at a sampling rate of 50 Hz using 30-s epochs. The actigraphic recordings will be analyzed with the MotionWare software (CamNtech Ltd., Cambridgeshire, England) to obtain indices of sleep quality (e.g., fragmentation index) and sleep quantity (e.g., total sleep time).

### Data-analytic plan

The collected data have a multilevel structure (i.e., repeated measurements nested within individuals). We will test our hypotheses with multilevel mixed-effects mediation analyses following principles and methods described in [174]. The statistical software package used is Mplus (see https://www.statmodel.com/). We expect a total of 840 usable data points for measures assessed once a day (sleep measures), 2520 data points for measures assessed three times a day (e.g., WTP), 3360 data points for measures assessed four times a day (e.g., mood), and 4200 data points for measures assessed five times a day (salivary parameters). Where appropriate, skewed variables will be transformed. We will use an alpha level of 0.05 for all tests. We will conduct sensitivity analyses by adding control variables to the models.

### Sample size calculation

The sample size calculation was performed with the support of a statistician. The power computations are based on a model by which the effect of WTP on each wellbeing and health-related outcome (Y) follows two paths, direct and indirect. In the indirect path, WTP acts on work-related workload (WL) and on work-related perseverative cognition (PC), which both act on Y. The computations rely on the repeated simulations of such model. The model and its assumptions are given in the Supplementary Material.

## Discussion

### Relevance and impact

Workplace telepressure is a recent concept in a quickly changing working world. It is essential to develop theory on how WTP may affect employees’ behavior, wellbeing, and health. The planned project will be the most comprehensive study to this day on short-term associations between within-person variations in WTP and important wellbeing and health-related experiential, physiological, and behavioral measures. The anticipated findings will greatly enhance our knowledge and understanding of WTP and thus help establish its relevance within the research domains of work, stress, and health. The carefully selected outcome measures are indicators of potential early-stage dysregulation of the allostatic processes. Investigating whether WTP is significantly associated with these indicators at the day level through the mediating role of work-related workload and work-related perseverative cognition is an important step towards understanding how high levels of WTP may lead in the long run to secondary alterations (e.g., hypertension, chronic inflammation, burnout) and disease (e.g., heart disease, clinical depression). The ambulatory assessment approach of the planned project using state-of-the-art methodological strategies is highly relevant as it allows for the investigation of employees’ subjective experience, physiology, and behavior in their everyday lives resulting in high ecological validity of the findings. High-quality ambulatory assessment studies are important to determine whether findings from studies in the setting of a laboratory also hold in more real-life environments.

As several measures of the proposed project represent potential intervention targets, we also anticipate the findings of the project to contribute significantly to guiding the development and implementation of theory-led interventions, programs, and policies. Such interventions aim at managing work-related demands and behaviors and favor employees’ wellbeing and health (e.g., sleep hygiene interventions, technology use education interventions at the organizational and personal level, perseverative cognition reduction interventions). Understanding the role of these potential intervention targets is necessary for accurate design and evaluation of effective interventions, programs, and policies. Moreover, the biobehavioral monitoring of the planned project consisting of salivary biomarkers, electrocardiographic measurement, and sleep actigraphy can add an important dimension to the evaluation of the effectiveness of such interventions, programs, and policies, which often relies on self-report measures only.

### Possible challenges

The planned project is the first to investigate relationships between WTP and several psychophysiological parameters in an ambulatory approach. Therefore, we are expecting possible challenges throughout the data collection period.

Firstly, some of our exclusion criteria (see Sect. "Participants") drastically affect the number of participants eligible for the ambulatory phase. For instance, sleep apnea is one of our exclusion criteria. Sleep apnea is a highly prevalent sleep disorder in the general adult population with an overall population prevalence ranging from 9 to 38% [175]. Similarly, approximately 120 000 persons suffer from this sleep-disordered breathing in the overall Swiss adult population, with 2% of middle-aged women and 4% of middle-aged men presenting at least five events per hour [176] and 23% of adult women and 50% of adult men presenting at least 15 events per hour in the Lausanne’s population where the present study is conducted. Additionally, we will exclude participants who take any medication that can affect the psychophysiological parameters of interest, in particular cardiac, salivary, and sleep parameters. For example, according to a Swiss health survey conducted in 2018, 18% of the Swiss adult population take hypertension medication [177]. As such, we might have to exclude a considerable number of participants from the ambulatory phase.

Secondly, we are aware that the study’s requirements facing participants are substantial. Given (1) the length of the participation period (i.e., seven consecutive days), (2) the number of daily sampling points (filling out questionnaires and saliva sampling four and five times per day, respectively) as well as the time spent on performing them, and (3) the burden of continuously wearing an ECG device throughout the seven-day participation period, we anticipate dropouts and non-compliance.

Lastly, we expect to deal with technical issues such as malfunctioning devices and software or hardware bugs. However, given that most participants will be recruited from nearby cities and surrounding areas, we are confident that participants would be able to promptly come to our laboratory in case we need to urgently fix a technical issue (e.g., unresponsive iPad, ECG monitor stops recording).

However, it is noteworthy that our research team managed to recruit 72 music students for a more demanding study, the pool of music students available in Switzerland being much smaller than the pool of employees using ICTs regularly [50]. The study was an ambulatory assessment in which Gomez and colleagues [50] measured physiological, experiential, and behavioral parameters during seven days. The participants were asked to fill in questionnaires and collect saliva samples six times throughout the day. The participants filled in the questionnaires with an iPod Touch using the software iDialogPad. The participants also wore the MotionWatch 8 and an electrocardiogram device. U.M. Nater performed the biochemical analyses of the saliva samples to determine sC and sAA. R. Heinzer and J. Haba-Rubio were consultants for the sleep-related part of the project. The questionnaires included the daily assessment of perseverative cognition, self-reported sleep duration and quality, mood, subjective health complaints, biobehavioral variables, and self-reported stressful events. Despite the challenges of this kind of research protocol, the team was able to achieve excellent success rates in terms of data acquisition. Over the seven days, participants had to answer 1533 items. In total, 95% of all answers were available for analysis. With regard to the salivary measures, participants had to collect 42 samples. For comparison, in the present study, participants will have to collect a total of 35 saliva samples (17% less). For sC and sAA, 95% and 92% of all samples were available for analysis, respectively. As to the actigraphic data, 85% of all possible data were available for analysis. This relatively low rate was due to a bug in the firmware of the MotionWatch 8 at the start of the study. We are therefore very confident that data collection of the present study will be highly successful.

Additionally, we have implemented several methods to face the challenges we might encounter.

Firstly, we have set different recruitment strategies that we will progressively deploy depending on the success rate of each strategy until reaching 120 participants with usable data. We will start by hanging our flyers in nearby universities and institutions and posting online versions on their respective websites to target the local and academic population. We will then publish a recruitment announcement in a local newspaper to target a much larger and more diverse population. This newspaper is delivered to over 100′000 readers in the Lausanne area. A final strategy will consist in sharing a recruitment announcement with the Human Resources of companies.

Secondly, during the laboratory visit, we will familiarize the participants with all the requirements of the study with the support of a PowerPoint presentation and information and instructions sheets. We will invite the participants to wear the Bittium Faros 180L and the MotionWatch 8 and perform saliva sampling to prepare them for the ambulatory phase. We will also go through all daily questionnaires using the same iPad they will bring home with them. All in all, the laboratory visit will allow the participants to know what the exact requirements of the ambulatory assessment are and, thus, decide whether they can comply satisfactorily with them. At the end of the laboratory visit, the participants should feel confident and ready for the ambulatory phase. Finally, to increase participants’ compliance, the investigator will explain to them that the financial remuneration will be proportional to their degree of compliance with the requirements. A 100% compliance (i.e., filling in all diaries and collecting all saliva samples at the defined times) will be rewarded with a 20% bonus per day.

Lastly, in order to minimize data loss, we will provide participants a troubleshooting sheet including all the information they would need to fix unexpected technical issues with the devices. We will also inform them that they can contact us throughout the ambulatory phase and that, should the need arise, they can either come to our laboratory or meet us at a mutually agreed upon location to come up with adequate solutions.

## Supplementary Information

**Additional file 1**. Questionnaires.

## Data Availability

Not applicable.

## Abbreviations

ECG: Electrocardiogram
HPA: Hypothalamic-pituitary-adrenal
HRV: Heart rate variability
ICT: Information and communication technology
PTP: Private life telepressure
sAA: Salivary alpha-amylase
SAM: Sympathoadrenal-medullary
sC: Salivary cortisol
sDHEA: Salivary dehydroepiandrosterone
WTP: Workplace telepressure
