# Supplementary material for: Does workplace telepressure get under the skin? Protocol for an ambulatory assessment study on wellbeing and health-related physiological, experiential, and behavioral concomitants of workplace telepressure
Source: BMC Psychol. 2023 May 3;11:145. doi: 10.1186/s40359-023-01123-4 (PMC10155671; doi:10.1186/s40359-023-01123-4)
Supplement: Supplementary file 1 — Additional file 1. Questionnaires. [file 40359_2023_1123_MOESM1_ESM.docx]

**Supplementary material of the study protocol “Does workplace telepressure get under the skin?**

**Protocol for an ambulatory assessment study on wellbeing and health-related physiological, experiential, and behavioral concomitants of workplace telepressure”**

**Table of contents**

[List of all questionnaires 2](#_Toc123737654)

[Supplementary Table 1: Online entry questionnaire measures 2](#_Toc123737655)

[Supplementary Table 2: Laboratory visit measures 3](#_Toc123737656)

[Supplementary Table 3: Ambulatory visit measures 4](#_Toc123737657)

[Full list of questions in French 6](#_Toc123737658)

[Questions of the online entry questionnaire 6](#_Toc123737659)

[Questions of the laboratory visit questionnaire 16](#_Toc123737660)

[Questions of the ambulatory assessment questionnaire (iDialogPad script) 31](#_Toc123737661)

[Sample size calculation 45](#_Toc123737662)

# List of all questionnaires

## Supplementary Table 1

Online entry questionnaire measures (assessed once)

| **Self-reported data** | **Scales (number of items)** |
| --- | --- |
| Sociodemographic data | Ad-hoc questions (23) |
| Health-related data | Ad-hoc questions (6)  The Insomnia Severity Index, Morin 1993 (7)  Alcohol Abuse/Dependence Module of the Patient Health Questionnaire, Spitzer et al. 2000 (6) |
| General WTP | The general WTP measure adapted from Barber & Santuzzi 2015 (6) |
| General PTP | The general PTP measure adapted from Barber & Santuzzi 2015 (6) |

## Supplementary Table 2

Laboratory visit measures (assessed once)

| **Self-reported data** | **Scales (number of items)** |
| --- | --- |
| Complementary sociodemographic and health-related data | Ad-hoc questions (15) |
| Workplace fear of missing out | The workplace Fear of Missing Out scale, Budnick et al. 2020 (10) |
| Psychological detachment from work | The psychological detachment subscale of the recovery experience questionnaire, Sonnentag & Fritz 2007 (4) |
| ICT-related response expectations and availability | Response expectations and availability subscales of the ICT Demand Scale, Day et al. 2012 (6) |
| Technostress creators | The technostress creators scale, Fuhrer 2021 (21) |
| Workaholism | The Dutch Work Addiction Scale, Schaufeli et al. 2009 (10) |
| Segmentation preferences and supplies | The workplace segmentation preferences and supplies measure, Kreiner 2006 (8) |
| Personality traits | “Neuroticism” and “Conscientiousness” dimensions of the Big Five Inventory, Plaisant et al. 2010 (17) |
| Depression, anxiety, and stress | The Depression, Anxiety and Stress Scale, Lovibond & Lovibond, 1995 (21) |
| Trait mindfulness | The Mindful Attention Awareness Scale, Jermann et al. 2009 (15) |
| General WTP | The general WTP measure adapted from Barber & Santuzzi 2015 (6) |
| General PTP | The general PTP measure adapted from Barber & Santuzzi 2015 (6) |

## Supplementary Table 3

Ambulatory assessment measures

| **Self-reported data** | **Scales (number of items)** | **Time of assessment** |
| --- | --- | --- |
| WTP | The WTP measure adapted from Barber & Santuzzi 2015 (6) | 12:30, 17:30, and bedtime (3 times) |
| PTP | The PTP measure adapted from Barber & Santuzzi 2015 (6) | 12:30, 17:30, and bedtime (3 times) |
| Work-related workload | The workload scale, Derks et al. 2014 (5) | 12:30, 17:30, and bedtime (3 times) |
| Private life workload | The private life workload scale, adapted from Derks et al. 2014 (3) | 12:30, 17:30, and bedtime (3 times) |
| Work-related perseverative cognition | The work-related worry/rumination measure, Flaxman et al. 2018 (4) | 12:30, 17:30, and bedtime (3 times) |
| Private life perseverative cognition | The private worry/rumination measure, adapted from Flaxman et al. 2018 (4) | 12:30, 17:30, and bedtime (3 times) |
| Number of stressful events | Number of stressful events experienced since the last assessment (1) | 12:30, 17:30, and bedtime (3 times) |
| Sleep | Self-reported sleep questionnaire (11) | 30 min after awakening |
| Biobehavioral measures | Number of units of caffeinated beverages, alcoholic beverages, tobacco products, e-cigarettes, and drugs (prescription/over-the-counter) taken since the last assessment (6) | +30 min, 12:30, 17:30, and bedtime (4 times) |
| Psychosomatic complaints | Somatic Symptom Scale-8, Gierk et al. 2014; Shirom-Melamed Burnout Measure, Sassi & Neveu 2010 (12) | +30 min, 12:30, 17:30, and bedtime (4 times) |
| Mood | The Multidimensional Mood State Questionnaire – short version, Wilhelm & Schoebi 2007 (8) | +30 min, 12:30, 17:30, and bedtime (4 times) |
| **Physiological measures** | **Type of assessment \| Instruments** |  |
| sC, sAA, and sDHEA | Saliva sampling \| SaliCaps (IBL International, Hamburg, Germany) | Awakening, +30 min, 12:30, 17:30, and bedtime (5 times) |
| Indices of HRV | ECG recording \| Bittium Faros 180L (Bittium Corporation, Oulu, Finland) | Continuous recording |
| Indices of sleep quality and quantity | Actigraphy \| MotionWatch 8 (CamNtech Ltd, Cambridgeshire, England) | Continuous recording |

The plus (+) sign in “+30 min” refers to “after awakening”.

# Full list of questions in French

## Questions of the online entry questionnaire

**Hello,**

**We invite you to answer all the following questions as honestly as possible. There are no right or wrong answers.**

**For confidentiality reasons, all the information you provide will be coded and anonymized. You can withdraw from the study at any time.**

**The following questionnaire will take approximately 15 minutes to complete.**

**Please click "Next" to begin the questionnaire.**

**There are 38 questions in this questionnaire.**

**1. Participation code**

Please enter the 3-digit participation code you received by e-mail.

Only numbers can be entered in this field.

Please enter your answer here: ___

**2. Consent**

Please check the "I agree" box to allow your data to be saved and processed for this study.

Please check the "I decline" box to stop the questionnaire and leave this page.

Please select only one of the following options:

- I accept
- I refuse

**3. Work-related questions**

Please check the days during which you work in a typical week.

For each day checked, please indicate whether you work in the morning, afternoon, or all day.

- Monday ____________________________________________
- Tuesday ____________________________________________
- Wednesday ____________________________________________
- Thursday ____________________________________________
- Friday ____________________________________________
- Saturday ____________________________________________
- Sunday ____________________________________________

**4. Average number of actual hours worked in your week (hours/week)**

Please enter your answer here: ___

**5.** **Frequency of use of information and communication technologies (e.g., email, smartphone, voicemail) for work-related communications only during the week/workdays**

Please select one of the following:

- All/Almost all workdays
- Some days
- Never

**6. Frequency of use of information and communication technologies (e.g., email, smartphone, voicemail) for work-related communications only on days off**

Please select one of the following:

- All/Almost all workdays
- Some days
- Never

**7. Work on weekend according to contract**

Please select one of the following:

- Yes
- No

**8. On-call hours according to contract (obligation to stay at your place of work/be available outside habitual working hours)**

Please select one of the following:

- Yes
- No

**9. Night shift (11 p.m. – 5 a.m.)**

Please select one of the following:

- Yes
- No

**10. Health-related questions**

Do you suffer from sleep apnea?

Sleep apnea is defined as a pause in breathing for at least 10 seconds during sleep.

Please select one of the following:

- Yes
- No
- I do not know

**11. Do you have any other disease(s) or medical condition(s) (e.g., cardiovascular, neurological, pulmonary, metabolic, autoimmune, and skin diseases, psychological disorders, sleep disorders, allergies, cancer)?**

Please select one of the following:

- Yes
- No

**12. Please list all diseases or medical conditions from which you are currently suffering.**

Please enter your answer here: ___

**13. Are you currently taking any prescription or non-prescription medications/products (including hormonal contraceptives or hormone replacement therapy)?**

Please select one of the following:

- Yes
- No

**14. Please list the exact name (according to package) of all prescription and non-prescription medications/products you are currently taking (including hormonal contraceptives or hormone replacement therapy).**

Please enter your answer here: ___

**15. Do you wear a pacemaker?**

Please select one of the following:

- Yes
- No

**16. Please rate the current (i.e., last month) severity of your insomnia problems:**

|  | 0 - None | 1 - Mild | 2 - Moderate | 3 - Severe | 4 - Very |
| --- | --- | --- | --- | --- | --- |
| Difficulty falling asleep |  |  |  |  |  |
| Difficulty staying asleep |  |  |  |  |  |
| Problem waking up too early |  |  |  |  |  |

**17. Select the appropriate answer for each item:**

|  | 0 – Very satisfied | 1 - Satisfied | 2 - Neutral | 3 - Dissatisfied | 4 – Very dissatisfied |
| --- | --- | --- | --- | --- | --- |
| How satisfied/dissatisfied are you with your current sleep pattern? |  |  |  |  |  |

**18. Select the appropriate answer for each item:**

|  | 0 - Not at all | 1 - A little | 2 - Somewhat | 3 - Much | 4 - Very much |
| --- | --- | --- | --- | --- | --- |
| To what extent do you consider your sleep problems to interfere with your daily functioning (e.g., daytime fatigue, ability to function at work/daily chores, concentration, memory, mood) |  |  |  |  |  |
| How noticeable to others do you think your sleeping problems are in terms of impairing the quality of your life? |  |  |  |  |  |
| How worried/distressed are you about your current sleep problems? |  |  |  |  |  |

**Usual waking time**

**19. In the past month, what time did you usually wake up in the morning on weekdays?**

Desired response format: hh:mm (e.g., 08:30)

Please enter your answer here: ___

**20. In the past month, what time did you usually wake up in the morning on the weekend?**

Desired response format: hh:mm (e.g., 08:30)

Please enter your answer here: ___

**Substance use**

**21. In the past 3 months, have you used one or more of the following products?**

Please select all that apply:

- Cigarettes
- E-cigarettes
- Cigars/cigarillos
- Hookahs (Shisha, waterpipe with tobacco only)
- Pipes (no water pipes/Hookahs)
- Snus ("plug", tobacco in pallet)
- Snuff (tobacco)
- Chewing tobacco
- I have not used any of these products.

**22. How many cigarettes do you usually smoke per week?**

Please enter your answer here: ___

**23. How many times a week do you usually use electronic cigarettes?**

Please enter your answer here: ___

**24. In the past 3 months, have you used any of the following substances?**

Please select all that apply:

- Cannabis
- Crack
- Poppers (volatile nitro derivatives)
- Amphetamines/Speed
- Methamphetamine (Crystal Meth/Tina)
- Cocaine
- Ecstasy/MDMA
- Mephedrone
- Other synthetic stimulants (e.g., MXE, bathsalts, 3MMC, 4MEC,4_FA, XTClight)
- Anabolic steroids (e.g., Testosterone)
- Opioids (e.g., Opium, Heroin, Morphine, Methadone)
- Hallucinogens (e.g., LSD, MDMA, PCP)
- Ketamine
- GHB/GBL
- Sedatives or tranquilizers (Valium, Rivotril, Rohypnol, Xanax, Seduxen, Phenazepam)
- I have taken substances that I do not know the name of.
- I have not used any of these substances.

**Alcohol consumption / abuse**

**25. Do you drink alcohol (including beer or wine)?**

Please select one of the following:

- Yes
- No

**26. Have any of the following happened to you more than once in the last six months?**

Select the appropriate answer for each item:

|  | Yes | No |
| --- | --- | --- |
| You drank alcohol even though a doctor suggested that you stop drinking because of a problem with your health. |  |  |
| You drank alcohol, were high from alcohol, or hung over while you were working, going to school, or taking care of children, or other responsibilities. |  |  |
| You missed or were late for work, school, or other activities because you were drinking or hung over. |  |  |
| You had a problem getting along with other people while you were drinking. |  |  |
| You drove a car after having several drinks or after drinking too much. |  |  |

**General workplace telepressure**

**27.** **For the following questions, think about how you use technology to communicate with people in your workplace. Specifically think about work-related message-based technologies that allow you to control when you respond (email, text messages, voicemail, etc.).**

Please rate how much you agree or disagree with the statements:

|  | 1 - Strongly disagree | 2 | 3 | 4 | 5 – Strongly agree |
| --- | --- | --- | --- | --- | --- |
| It’s hard for me to focus on other things when I receive a work-related message from someone. |  |  |  |  |  |
| I can concentrate better on other tasks once I’ve responded to my work-related messages. |  |  |  |  |  |
| I can’t stop thinking about a work-related message until I’ve responded. |  |  |  |  |  |
| I feel a strong need to respond to others immediately. |  |  |  |  |  |
| I have an overwhelming feeling to respond right at that moment when I receive a work-related request from someone. |  |  |  |  |  |
| It’s difficult for me to resist responding to a work-related message right away. |  |  |  |  |  |

**General private life telepressure**

**28. For the following questions, think about how you use technology to communicate with people in your private life. Specifically think about message-based technologies that allow you to control when you respond (email, text messages, voicemail, etc.) in your private life.**

Please rate how much you agree or disagree with the statements:

|  | 1 – Strongly disagree | 2 | 3 | 4 | 5 – Strongly agree |
| --- | --- | --- | --- | --- | --- |
| It’s hard for me to focus on other things when I receive a personal message from someone. |  |  |  |  |  |
| I can concentrate better on other tasks once I’ve responded to my personal messages. |  |  |  |  |  |
| I can’t stop thinking about a personal message until I’ve responded. |  |  |  |  |  |
| I feel a strong need to respond to others immediately. |  |  |  |  |  |
| I have an overwhelming feeling to respond right at that moment when I receive a personal request from someone. |  |  |  |  |  |
| It’s difficult for me to resist responding to a personal message right away. |  |  |  |  |  |

**General questions**

**29. Last name**

Please enter your answer here: ___

**30. First name**

Please enter your answer here: ___

**31. Age (e.g., 25)**

Please enter your answer here: ___

**32. Height in centimeters (e.g., 170)**

Please enter your answer here: ___

**33. Weight in kilograms (p. ex. 70)**

Please enter your answer here: ___

**34. Mother tongue**

Please enter your answer here: ___

**35.** **How do you identify?**

Please select one of the following:

- As a woman
- As a man
- As a transgender person
- Other

**36. Are you currently pregnant?**

Please select one of the following:

- Yes
- No

**37. Are you currently breastfeeding?**

Please select one of the following:

- Yes
- No

**38. French skills**

1: I do not speak or understand French.

5: I speak and understand French very well.

Please select one of the following:

- 1
- 2
- 3
- 4
- 5

**Thank you for your answers.**

**If you have any questions or remarks, you can contact us by e-mail (example@gmail.com) or by phone (021 123 45 67).**

**Goodbye.**

## Questions of the laboratory visit questionnaire

**Hello,**

**First, we would like to thank you for your interest in our study that aims to study the links between telepressure at work, wellbeing, and health.**

**For confidentiality reasons, all the information you provide will be coded and anonymized. You can withdraw from the study at any time.**

**The following questionnaire will take you approximately 15 minutes to complete.**

**Please go to the next page to begin the questionnaire.**

**There are 28 questions in this questionnaire.**

**1. Participation code**

Please enter the 3-digit participation code you received by e-mail.

Only numbers can be entered in this field.

Please enter your answer here: ___

**2. Consent**

Please check the "I agree" box to allow your data to be saved and processed for this study.

Please check the "I decline" box to stop the questionnaire and leave this page.

Please select only one of the following options:

- I accept
- I refuse

**General questions**

**3. How do you identify?**

Please select one of the following:

- As a woman
- As a man
- As a transgender person
- Other

**4. Do you use hormonal contraceptives?**

Please select one of the following:

- Yes
- No

**5. Are you on hormone replacement therapy?**

Please select one of the following:

- Yes
- No

**6. What is the typical average length of your menstrual cycle (i.e., from the first day of your period to the first day of your next period)? Please indicate the number of days. If you do not have a menstrual cycle, please enter « None ».**

Please enter your answer here: ___

**7. What is your typical average period length? Please indicate the number of days.**

**If you have not had a period, please indicate "None".**

Please enter your answer here: ___

**8. When was your last period? Please indicate the day and month.**

**If you do not have a period, please indicate "None".**

Please enter your answer here: ___

**9. Marital status**

Please select one of the following:

- Single
- Married
- Concubinage
- Divorced
- Widowed

**10. Number of children under the age of 18 currently living in the household:**

Please enter your answer here: ___

**11. Total number of adults living in the same household:**

Please enter your answer here: ___

**12. Total number of individuals living in the same household:**

Please enter your answer here: ___

**13. Please check the box that corresponds to your highest level of education/training.**

Please select one of the following:

- Nongraduate
- School
- High school
- Vocational training
- Higher education

**14. Please indicate the name of your profession:**

Please enter your answer here: ___

**15. Please indicate the percentage corresponding to the place you work in a typical week. The total should be 100 (e.g., at work: 75, at home/telecommuting: 20, other location(s): 5).**

- At work
- At home / telecommuting
- Other place(s)

**16. Seniority (number of years since you started your current job)**

Please enter your answer here: ___

**17. Do you have a management or supervisory role (e.g., team management)?**

Please select one of the following:

- Yes
- No

**Personality traits**

**18. Here are a number of characteristics that may or may not apply to you. For example, do you agree that you are someone who likes to spend time with others? Please write a number next to each statement to indicate the extent to which you agree or disagree with that statement.**

**I see myself as someone who…**

|  | 1 – Disagree strongly | 2 – Disagree a little | 3 – Neither agree nor disagree | 4 – Agree a little | 5 – Agree strongly | |
| --- | --- | --- | --- | --- | --- | --- |
| Is depressed, blue |  |  |  |  |  |  |
| Is relaxed, handles stress well |  |  |  |  |  |  |
| Can be tense |  |  |  |  |  |  |
| Worries a lot |  |  |  |  |  |  |
| Is emotionally stable, not easily upset |  |  |  |  |  |  |
| Can be moody |  |  |  |  |  |  |
| Remains calm in tense situations |  |  |  |  |  |  |
| Gets nervous easily |  |  |  |  |  |  |
| Does a thorough job |  |  |  |  |  |  |
| Can be somewhat careless |  |  |  |  |  |  |
| Is a reliable worker |  |  |  |  |  |  |
| Tends to be disorganized |  |  |  |  |  |  |
| Tends to be lazy |  |  |  |  |  |  |
| Perseveres until the task is finished |  |  |  |  |  |  |
| Does things efficiently |  |  |  |  |  |  |
| Makes plans and follows through with them |  |  |  |  |  |  |
| Is easily distracted |  |  |  |  |  |  |

**Depression, anxiety, and stress**

**19. Please read each statement and indicate which one best describes your experience over the past week. Indicate your choice by checking the corresponding number (0, 1, 2 or 3). There are no right or wrong answers. Do not spend too much time on the statements.**

|  | 0 – Does not apply to me | 1 – Applies to me a little, or some of the time | 2 – Applies to me a lot, or a good part of the time | 3 – Applies to me entirely, or the vast majority of the time |
| --- | --- | --- | --- | --- |
| I found it hard to wind down. |  |  |  |  |
| I was aware of dryness of my mouth. |  |  |  |  |
| I couldn’t seem to experience any positive feeling at all. |  |  |  |  |
| I experienced breathing difficulty (e.g., excessively rapid breathing, breathlessness in the absence of physical exertion). |  |  |  |  |
| I found it difficult to work up the initiative to do things. |  |  |  |  |
| I tended to over-react to situations. |  |  |  |  |
| I experienced trembling (e.g., in the hands). |  |  |  |  |
| I felt that I was using a lot of nervous energy. |  |  |  |  |
| I was worried about situations in which I might panic and make a fool of myself. |  |  |  |  |
| I felt that I had nothing to look forward to. |  |  |  |  |
| I found myself getting agitated. |  |  |  |  |
| I found it difficult to relax. |  |  |  |  |
| I felt downhearted and blue. |  |  |  |  |
| I was intolerant of anything that kept me from getting on with what I was doing. |  |  |  |  |
| I felt I was close to panic. |  |  |  |  |
| I was unable to become enthusiastic about anything. |  |  |  |  |
| I felt I wasn’t worth much as a person. |  |  |  |  |
| I felt that I was rather touchy. |  |  |  |  |
| I was aware of the action of my heart in the absence of physical exertion (e.g., sense of heart rate increase, heart missing a beat). |  |  |  |  |
| I felt scared without any good reason. |  |  |  |  |
| I felt that life was meaningless. |  |  |  |  |

**Trait mindfulness**

**20. Below is a collection of statements about your everyday experience. Using the 1-6 scale below, please indicate how frequently or infrequently you currently have each experience. Please answer according to what really reflects your experience rather than what you think your experience should be. Please treat each item separately from every other item.**

|  | 1 – Almost always | 2 – Very frequently | 3 – Somewhat frequently | 4 – Somewhat infrequently | 5 – Very frequently | 6 – Almost never |
| --- | --- | --- | --- | --- | --- | --- |
| I could be experiencing some emotion and not be conscious of it until some time later. |  |  |  |  |  |  |
| I break or spill things because of carelessness, not paying attention, or thinking of something else. |  |  |  |  |  |  |
| I find it difficult to stay focused on what’s happening in the present. |  |  |  |  |  |  |
| I tend to walk quickly to get where I’m going without paying attention to what I experience along the way. |  |  |  |  |  |  |
| I tend not to notice feelings of physical tension or discomfort until they really grab my attention. |  |  |  |  |  |  |
| I forget a person’s name almost as soon as I’ve been told it for the first time. |  |  |  |  |  |  |
| It seems I am “running on automatic,” without much awareness of what I’m doing. |  |  |  |  |  |  |
| I rush through activities without being really attentive to them. |  |  |  |  |  |  |
| I get so focused on the goal I want to achieve that I lose touch with what I’m doing right now to get there. |  |  |  |  |  |  |
| I do jobs or tasks automatically, without being aware of what I'm doing. |  |  |  |  |  |  |
| I find myself listening to someone with one ear, doing something else at the same time. |  |  |  |  |  |  |
| I drive places on ‘automatic pilot’ and then wonder why I went there. |  |  |  |  |  |  |
| I find myself preoccupied with the future or the past. |  |  |  |  |  |  |
| I find myself doing things without paying attention. |  |  |  |  |  |  |
| I snack without being aware that I’m eating. |  |  |  |  |  |  |

**General workplace telepressure**

**21. For the following questions, think about how you use technology to communicate with people in your workplace. Specifically think about work-related message-based technologies that allow you to control when you respond (email, text messages, voicemail, etc.).**

Please rate how much you agree or disagree with the statements:

|  | 1 – Strongly disagree | 2 | 3 | 4 | 5 – Strongly agree |
| --- | --- | --- | --- | --- | --- |
| It’s hard for me to focus on other things when I receive a work-related message from someone. |  |  |  |  |  |
| I can concentrate better on other tasks once I’ve responded to my work-related messages. |  |  |  |  |  |
| I can’t stop thinking about a work-related message until I’ve responded. |  |  |  |  |  |
| I feel a strong need to respond to others immediately. |  |  |  |  |  |
| I have an overwhelming feeling to respond right at that moment when I receive a work-related request from someone. |  |  |  |  |  |
| It’s difficult for me to resist responding to a work-related message right away. |  |  |  |  |  |

**General private life telepressure**

**22. For the following questions, think about how you use technology to communicate with people in your private life. Specifically think about message-based technologies that allow you to control when you respond (email, text messages, voicemail, etc.) in your private life.**

Please rate how much you agree or disagree with the statements:

|  | 1 – Strongly disagree | 2 | 3 | 4 | 5 – Strongly agree |
| --- | --- | --- | --- | --- | --- |
| It’s hard for me to focus on other things when I receive a personal message from someone. |  |  |  |  |  |
| I can concentrate better on other tasks once I’ve responded to my personal messages. |  |  |  |  |  |
| I can’t stop thinking about a personal message until I’ve responded. |  |  |  |  |  |
| I feel a strong need to respond to others immediately. |  |  |  |  |  |
| I have an overwhelming feeling to respond right at that moment when I receive a personal request from someone. |  |  |  |  |  |
| It’s difficult for me to resist responding to a personal message right away. |  |  |  |  |  |

**Technostress**

**23. Technostress relates to the phenomenon of stress experienced by end users in organizations as a result of their use of Information and Communication Technologies (ICTs). ICTs can be defined as all techniques and devices that allow for remote electronic communication (e.g., smartphones, email, calls, applications). Therefore, the creators of technostress or "technostressors" are the factors that create the stress caused by ICT use.**

**In this questionnaire, you are asked to evaluate how technostressors affect your professional and private life. Please read the following statements and indicate how they apply to you.**

|  | 1 – Strongly disagree | 2 | 3 | 4 | 5 | 6 | 7 – Strongly agree |
| --- | --- | --- | --- | --- | --- | --- | --- |
| I am forced by this technology to work much faster. |  |  |  |  |  |  |  |
| I am forced by this technology to do more work than I can handle. |  |  |  |  |  |  |  |
| I am forced by this technology to work with very tight time schedules. |  |  |  |  |  |  |  |
| I have a higher workload because of increased technology complexity. |  |  |  |  |  |  |  |
| I spend less time with my family due to this technology. |  |  |  |  |  |  |  |
| I have to be in touch with my work even during my vacation due to this technology. |  |  |  |  |  |  |  |
| I have to sacrifice my vacation and weekend time to keep current on new technologies. |  |  |  |  |  |  |  |
| I feel my personal life is being invaded by this technology. |  |  |  |  |  |  |  |
| I do not know enough about this technology to handle my job satisfactorily. |  |  |  |  |  |  |  |
| I need a long time to understand and use new technologies. |  |  |  |  |  |  |  |
| I do not find enough time to study and upgrade my technology skills. |  |  |  |  |  |  |  |
| I often find it too complex for me to understand and use new technologies. |  |  |  |  |  |  |  |
| I feel constant threat to my job security due to new technologies. |  |  |  |  |  |  |  |
| I have to constantly update my skills to avoid being replaced. |  |  |  |  |  |  |  |
| I am threatened by coworkers with newer technology skills. |  |  |  |  |  |  |  |
| I do not share my knowledge with my coworkers for fear of being replaced. |  |  |  |  |  |  |  |
| I feel there is less sharing of knowledge among coworkers for fear of being replaced. |  |  |  |  |  |  |  |
| There are always new developments in the technologies we use in our organization. |  |  |  |  |  |  |  |
| There are constant changes in computer software in our organization. |  |  |  |  |  |  |  |
| There are constant changes in computer hardware in our organization. |  |  |  |  |  |  |  |
| There are frequent upgrades in computer networks in our organization. |  |  |  |  |  |  |  |

**Response expectations and availability**

**24. Please indicate how often you encounter each of the following situations at work.**

Choose the appropriate answer for each item:

|  | 0 - Never | 1 | 2 | 3 | 4 – Almost always |
| --- | --- | --- | --- | --- | --- |
| I am expected to respond to e-mail messages immediately. |  |  |  |  |  |
| I am expected to respond to voicemail messages immediately. |  |  |  |  |  |
| I am expected to be accessible at all times (e.g., through pager, cell phone, instant messaging). |  |  |  |  |  |
| Technology enables people I work with to contact me at any time. |  |  |  |  |  |
| I’m expected to check e-mail and/or voicemail when I’m out of the office. |  |  |  |  |  |
| I’m contacted about work-related issues outside of regular work hours. |  |  |  |  |  |

**Psychological detachment**

**25. Here are different statements that describe how you behave after work, on weekends, or during your free time.**

**Please indicate how much you agree with each statement when thinking about your free time.**

|  | 1 – I do not agree at all | 2 | 3 | 4 | 5 – I fully agree |
| --- | --- | --- | --- | --- | --- |
| I forget about work. |  |  |  |  |  |
| I don’t think about work at all. |  |  |  |  |  |
| I distance myself from my work. |  |  |  |  |  |
| I get a break from the demands of work. |  |  |  |  |  |

**Fear of Missing Out (FoMO)**

**26. Please indicate your agreement with each statement while thinking of how you typically feel or feel on average when away (e.g., off duty) or disconnected (e.g., not available via email, text, or instant messaging devices) from work.**

**When I am absent or disconnected from work:**

|  | 1 – Strongly disagree | 2 - Disagree | 3 – Neither agree nor disagree | 4 - Agree | 5 – Strongly agree |
| --- | --- | --- | --- | --- | --- |
| I worry that I will miss out on networking opportunities that my coworkers will have. |  |  |  |  |  |
| I am constantly thinking that I might miss opportunities to make new business contacts. |  |  |  |  |  |
| I am constantly thinking that I might miss opportunities to strengthen business contacts. |  |  |  |  |  |
| I fear that my coworkers might make business contacts that I won’t make. |  |  |  |  |  |
| I get anxious that I will miss out on an opportunity to make important business connections. |  |  |  |  |  |
| I worry that I might miss out on valuable work-related information. |  |  |  |  |  |
| I worry that I will miss out on important information that is relevant to my job. |  |  |  |  |  |
| I worry that I might miss important work-related updates. |  |  |  |  |  |
| I worry I will not know what is happening at work. |  |  |  |  |  |
| I worry that I will miss out on important work-related news. |  |  |  |  |  |

**Workaholism**

**27. Please read the following statements and indicate how they apply to you.**

Choose the appropriate answer for each item:

|  | 1 - Never | 2 | 3 | 4 - Always |
| --- | --- | --- | --- | --- |
| I seem to be in a hurry and racing against the clock. |  |  |  |  |
| I find myself continuing work after my co-workers have called it quits. |  |  |  |  |
| I stay busy and keep my irons in the fire. |  |  |  |  |
| I spend more time working than socializing with friends, on hobbies, or on leisure activities. |  |  |  |  |
| I find myself doing two or three things at one time such as eating lunch and writing a memo, while talking on the phone. |  |  |  |  |
| It’s important for me to work hard even when I don’t enjoy what I’m doing. |  |  |  |  |
| I often feel that there’s something inside me that drives me to work hard. |  |  |  |  |
| I feel obliged to work hard, even when it’s not enjoyable. |  |  |  |  |
| I feel guilty when I take time off work. |  |  |  |  |
| It is hard for me to relax when I’m not working. |  |  |  |  |

**Segmentation preferences and supplies**

**28. Segmentation is defined as the degree to which aspects of each domain (such as thoughts, concerns, physical markers) are kept separate from one another – cognitively, physically, or behaviorally). Segmentation supplies refer to the degree to which the organization in which the individual performs his or her job allows him or her to separate his or her private and professional life.**

Please read the following statements and indicate how they apply to you.

|  | 1 – Strongly disagree | 2 | 3 | 4 - Neutral | 5 | 6 | 7 – Strongly agree |
| --- | --- | --- | --- | --- | --- | --- | --- |
| I don’t like to have to think about work while I’m at home. |  |  |  |  |  |  |  |
| I prefer to keep work life at work. |  |  |  |  |  |  |  |
| I don’t like work issues creeping into my home life. |  |  |  |  |  |  |  |
| I like to be able to leave work behind when I go home. |  |  |  |  |  |  |  |
| My workplace lets people forget about work when they’re at home. |  |  |  |  |  |  |  |
| Where I work, people can keep work matters at work. |  |  |  |  |  |  |  |
| At my workplace, people are able to prevent work issues from creeping into their home life. |  |  |  |  |  |  |  |
| Where I work, people can mentally leave work behind when they go home. |  |  |  |  |  |  |  |

**Thank you for your answers.**

**If you have any questions or remarks, you can contact us by e-mail (example@gmail.com) or by phone (021 123 45 67).**

**Goodbye.**

## Questions of the ambulatory assessment questionnaire

**iDialogPad script (on provided iPads)**

SAN debug

GOP 2+128

OPT 3

PSEL 99

TOUT 600

PTOUT 0

EVENT 12:30 " Reminder: it's time for the midday questionnaire!" 4 1

EVENT 17:30 " Reminder: it's time for the afternoon questionnaire!" 4 1

PRINT "Hello!\r\rImportant: do not leave the application open for more than 10 minutes without touching the screen, in which case it will close."

PRINT " First, swallow the saliva in your mouth."

GTS "@saliva_start@"

PRINT " Now hold your saliva in your mouth for two minutes. You can no longer swallow and must then transfer the collected saliva into the tube. Press OK to start the timer."

OPT 3+512

PTOUT 10

PRINT "Please do not swallow, 120 seconds left..."

PRINT " Please do not swallow, 110 seconds left…"

PRINT "Please do not swallow, 100 seconds left…"

PRINT "Please do not swallow, 90 seconds left…"

PRINT "Please do not swallow, 80 seconds left…"

PRINT "Please do not swallow, 70 seconds left…"

PRINT "Please do not swallow, 60 seconds left…"

PRINT "Please do not swallow, 50 seconds left…"

PRINT "Please do not swallow, 40 seconds left…"

PRINT "Please do not swallow, 30 seconds left…"

PRINT "Please do not swallow, 20 seconds left…"

PTOUT 1

PRINT "10 seconds to go…"

PRINT "9 seconds to go…."

PRINT "8 seconds to go…"

PRINT "7 seconds to go…"

PRINT "6 seconds to go…"

PRINT "5 seconds to go…"

PRINT "4 seconds to go…"

PRINT "3 seconds to go…"

PRINT "2 seconds to go…"

PRINT "1 second to go…"

OPT 3

PTOUT 0

PRINT " Now transfer the saliva into the tube using the straw. Make sure you have closed the tube on all sides."

GTS "@saliva_end@"

FSIZ 20

ASK1 "@q000@ Were you able to follow the instructions for saliva collection? The instructions are the following: for the last 30 minutes, do not drink (other than water), eat, smoke, or engage in strenuous physical activity; and for the last 60 minutes, do not brush your teeth." 2 "Yes" "No"

SRE 1: intro

SRE 2: back

:back

IFSIZ 24

INPUT "@q000b@What was the problem?" 30+4

:intro

INPUT "@q001@Now please enter the number indicated on the SaliCap." 30+4

ASK1 "@q002@ Now please enter the time of day indicated on the SaliCap." 5 "1. Awakening" "2. Awakening+30min" "3. 12h30" "4. 17h30" "5. Bedtime"

SRE 1: Q01

SRE 2: Q02

SRE 3: Q03

SRE 4: Q03

SRE 5: Q03

:Q01

FSIZ 24

TOUT 600

PRINT "Merci!\r\rYou must now set a 30 minute timer for the second saliva collection.\r\rDon't forget to press the center button on your watch (MotionWatch 8) when you get up.\r\rNow press OK and exit the application."

SKIP: end

OPT 3

:Q02

FSIZ 24

PRINT " When you are ready to start the questionnaires, press on OK."

GTS "@quest_start@"

PRINT " This questionnaire is about your sleep during the last 24 hours."

TIMS "@sleepq01@What time did you turn off the light to sleep?" 1

TIMS "@sleepq02@How long did it take you to fall asleep?" 4

TIMS "@sleepq03@What time did you wake up (for the last time) this morning?" 1

TIMS "@sleepq04@What time did you get up (for the last time) this morning? Please indicate 00:00 if you are still in bed." 1

TIMS "@sleepq05@In total, how much sleep did you get last night?" 4

ASK1 "@sleepq06@This morning, did you wake up spontaneously/naturally?" 2 "Yes" "No"

SCALE "1 2 3 4 5 6" "Very bad" "Very good"

ASK2 "@sleepq07@How was your sleep?"

SCALE "1 2 3 4 5 6" "Very restless" "Very calm"

ASK2 "@sleepq08@How restless/calm was your sleep?"

SCALE "1 2 3 4 5 6" "Very difficult" "Very easy"

ASK2 "@sleepq09@How difficult/easy did you find it to fall asleep?"

SCALE "1 2 3 4 5 6" "Extremely bothered" "Not at all bothered"

ASK2 "@sleepq10@To what extent have you been bothered by waking up during the night?"

SCALE "1 2 3 4 5 6" "Extremely bothered" "Not at all bothered"

ASK2 "@sleepq11@How bothered were you by a premature awakening this morning?"

SKIP: BIO

:Q03

PRINT " For the following questions, think about how you have used technology to communicate with people in your workplace since the last questionnaire (the one you were supposed to complete at the predefined time). Specifically, think about message-based technologies that allow you to control when you respond (email, text messages, voicemail, etc.).\r\rPlease indicate the extent to which you agree or disagree with the following statements."

:WTPQ01

SCALE "1 2 3 4 5 6" "Strongly disagree" "Strongly agree"

ASK2 "@wtpq01@It was hard for me to focus on other things when I received a work-related message from someone."

:WTPQ02

SCALE "1 2 3 4 5 6" "Strongly disagree" "Strongly agree"

ASK2 "@wtpq02@I could concentrate better on other tasks once I’ve responded to my work-related messages."

:WTPQ03

SCALE "1 2 3 4 5 6" "Strongly disagree" "Strongly agree"

ASK2 "@wtpq03@I couldn’t stop thinking about a work-related message until I’ve responded."

:WTPQ04

SCALE "1 2 3 4 5 6" "Strongly disagree" "Strongly agree"

ASK2 "@wtpq04@I felt a strong need to respond to others immediately."

:WTPQ05

SCALE "1 2 3 4 5 6" "Strongly disagree" "Strongly agree"

ASK2 "@wtpq05@I had an overwhelming feeling to respond right at that moment when I received a work-related request from someone."

:WTPQ06

SCALE "1 2 3 4 5 6" "Strongly disagree" "Strongly agree"

ASK2 "@wtpq06@It was difficult for me to resist responding to a work-related message right away."

PTOUT 0

PRINT "For the following questions, think about how you have used technology to communicate with people in your personal life since the last questionnaire. Specifically, think about message-based technologies that allow you to control when you respond (email, text messages, voice mail, etc.).\r\rPlease indicate the extent to which you agree or disagree with the following statements."

:PTPQ01

SCALE "1 2 3 4 5 6" "Strongly disagree" "Strongly agree"

ASK2 "@ptpq01@It was hard for me to focus on other things when I received a personal message from someone."

:PTPQ02

SCALE "1 2 3 4 5 6" "Strongly disagree" "Strongly agree"

ASK2 "@ptpq02@I could concentrate better on other tasks once I’ve responded to my personal messages."

:PTPQ03

SCALE "1 2 3 4 5 6" "Strongly disagree" "Strongly agree"

ASK2 "@ptpq03@I couldn’t stop thinking about a personal message until I’ve responded."

:PTPQ04

SCALE "1 2 3 4 5 6" "Strongly disagree" "Strongly agree"

ASK2 "@ptpq04@I felt a strong need to respond to others immediately."

:PTPQ05

SCALE "1 2 3 4 5 6" "Strongly disagree" "Strongly agree"

ASK2 "@ptpq05@I had an overwhelming feeling to respond right at that moment when I received a personal request from someone."

:PTPQ06

SCALE "1 2 3 4 5 6" "Strongly disagree" "Strongly agree"

ASK2 "@ptpq06@It was difficult for me to resist responding to a personal message right away."

:COGQ01

FSIZ 18

PRINT "Perseverative cognition at work is defined as any repetitive thought or mental image about past, present, or future problems, concerns, events, experiences, or stressful situations related to your work." 4

TIMS "@cogq01@What is the total duration of your work-related perseverative cognition (in hours and minutes) since the last questionnaire?" 4

FSIZ 18

PRINT "Private life perseverative cognition is defined as any repetitive thought or mental image about past, present, or future problems, concerns, events, experiences, or stressful situations related to your private life." 4

TIMS "@cogq01b@What is the total duration of your private life perseverative cognition (in hours and minutes) since the last questionnaire?" 4

:COGQ02

FSIZ 24

SCALE "1 2 3 4 5 6" "Not at all" "A lot"

ASK2 "@cogq02@Since the last questionnaire:\rI worried about things I need to do at work."

:COGQ03

SCALE "1 2 3 4 5 6" "Not at all" "A lot"

ASK2 "@cogq03@Since the last questionnaire:\rI worried about how I would deal with a work task or issue."

:COGQ04

SCALE "1 2 3 4 5 6" "Not at all" "A lot"

ASK2 "@cogq04@Since the last questionnaire:\rMy thoughts kept returning to a stressful situation at work."

:COGQ05

SCALE "1 2 3 4 5 6" "Not at all" "A lot"

ASK2 "@cogq05@Since the last questionnaire:\rI worried about things I need to do at home/in my private life."

:COGQ06

SCALE "1 2 3 4 5 6" "Not at all" "A lot"

ASK2 "@cogq06@Since the last questionnaire:\rI worried about how I would deal with a task or issue at home/in my private life."

:COGQ07

SCALE "1 2 3 4 5 6" "Not at all" "A lot"

ASK2 "@cogq07@Since the last questionnaire:\rMy thoughts kept returning to a stressful situation at home/in my private life."

:WORKQ01

TIMS "@workq01@How much time (in hours and minutes) have you spent on your work tasks since the last questionnaire?" 4

:WORKQ02

IFSIZ 18

INPUT "@workq02@In which location(s) (work, home/telecommuting, other location(s)) have you performed these work tasks since the last questionnaire?\r\rPlease indicate the percentage corresponding to each location by entering *W* for *work*,*H* for *home/telecommuting*, and *O* for *other location(s)*, e.g.: W50 H50 O0" 30+4

IFSIZ 24

FSIZ 24

PRINT "To answer the following 3 statements, please refer only to the work activities you have performed since the last questionnaire."

:WORKQ03

SCALE "1 2 3 4 5 6" "Strongly disagree" "Strongly agree"

ASK2 "@workq03@I had too much work to do."

:WORKQ04

SCALE "1 2 3 4 5 6" "Strongly disagree" "Strongly agree"

ASK2 "@workq04@I had to work extra hard to get things done."

:WORKQ05

SCALE "1 2 3 4 5 6" "Strongly disagree" "Strongly agree"

ASK2 "@workq05@I had to work very quickly"

PRINT "To answer the following 3 statements, please refer only to activities at home / in your private life that you have done since the last questionnaire."

:WORKQ06

SCALE "1 2 3 4 5 6" "Strongly disagree" "Strongly agree"

ASK2 "@workq06@I had too much work to do."

:WORKQ07

SCALE "1 2 3 4 5 6" "Strongly disagree" "Strongly agree"

ASK2 "@workq07@I had to work extra hard to get things done."

:WORKQ08

SCALE "1 2 3 4 5 6" "Strongly disagree" "Strongly agree"

ASK2 "@workq08@I had to work very quickly."

FSIZ 18

ASK1 "@stresq01@How many stressful events have you experienced since the last questionnaire? Stressful events are minor and major events that have made you feel tense, irritated, angry, sad, disappointed, or that have made you feel a negative feeling or emotion of some other kind." 16 "0" "1" "2" "3" "4" "5" "6" "7" "8" "9" "10" "11" "12" "13" "14" "15"

:BIO

FSIZ 24

ASK1 "@bioq01@Have you taken any medication(s) since the last questionnaire?" 2 "Yes" "No"

SRE 1 :yes

SRE 2 :no

:yes

IFSIZ 24

INPUT "@bioq02@Please indicate the exact and complete name of the medication (according to the packaging)." 30+4

:no

ASK1 "@bioq03@How many alcoholic beverages (including beer and wine) have you consumed since the last questionnaire?" 16 "0" "1" "2" "3" "4" "5" "6" "7" "8" "9" "10" "11" "12" "13" "14" "15"

ASK1 "@bioq04@How many tobacco products (e.g., cigarettes, cigars, chewing tobacco) have you used since the last questionnaire?" 16 "0" "1" "2" "3" "4" "5" "6" "7" "8" "9" "10" "11" "12" "13" "14" "15"

ASK1 "@bioq05@How many electronic cigarettes have you consumed since the last questionnaire?" 16 "0" "1" "2" "3" "4" "5" "6" "7" "8" "9" "10" "11" "12" "13" "14" "15"

ASK1 "@bioq06@How many psychotropic drugs (e.g., cannabis, amphetamines, anabolic steroids) have you consumed since the last questionnaire?" 16 "0" "1" "2" "3" "4" "5" "6" "7" "8" "9" "10" "11" "12" "13" "14" "15"

PTOUT 0

PRINT "At this moment, how much are you bothered by any of the following problems?"

SCALE "1 2 3 4 5 6" "Not at all" "Extremely"

ASK2 "@psyq01@I am having stomach or bowel problems"

SCALE "1 2 3 4 5 6" "Not at all" "Extremely"

ASK2 "@psyq02@I am feeling back pain."

SCALE "1 2 3 4 5 6" "Not at all" "Extremely"

ASK2 "@psyq03@I am feeling pain in my arms, legs, or joints."

SCALE "1 2 3 4 5 6" "Not at all" "Extremely"

ASK2 "@psyq04@I am having headaches."

SCALE "1 2 3 4 5 6" "Not at all" "Extremely"

ASK2 "@psyq05@I am feeling chest pain or shortness of breath."

SCALE "1 2 3 4 5 6" "Not at all" "Extremely"

ASK2 "@psyq06@I am feeling dizzy."

SCALE "1 2 3 4 5 6" "Not at all" "Extremely"

ASK2 "@psyq07@I am feeling tired or having low energy."

SCALE "1 2 3 4 5 6" "Not at all" "Extremely"

ASK2 "@psyq08@My thinking process is slow."

SCALE "1 2 3 4 5 6" "Not at all" "Extremely"

ASK2 "@psyq09@I have difficulty concentrating."

SCALE "1 2 3 4 5 6" "Not at all" "Extremely"

ASK2 "@psyq10@I feel I am not thinking clearly."

SCALE "1 2 3 4 5 6" "Not at all" "Extremely"

ASK2 "@psyq11@I feel I am not focused on my thinking."

SCALE "1 2 3 4 5 6" "Not at all" "Extremely"

ASK2 "@psyq12@I have difficulty thinking about complex things."

SCALE "1 2 3 4 5 6 7 8 9" "Extremely unwell" "Extremely well"

ASK2 "@humq01@At this moment, I am/feel:"

SCALE "1 2 3 4 5 6 7 8 9" "Extremely relaxed" "Extremely tense"

ASK2 "@humq02@At this moment, I am/feel:"

SCALE "1 2 3 4 5 6 7 8 9" "Extremely tired" "Extremely awake"

ASK2 "@humq03@At this moment, I am/feel:"

SCALE "1 2 3 4 5 6 7 8 9" "Extremely content" "Extremely discontent"

ASK2 "@humq04@At this moment, I am/feel:"

SCALE "1 2 3 4 5 6 7 8 9" "Extremely agitated" "Extremely calm"

ASK2 "@humq05@At this moment, I am/feel:"

SCALE "1 2 3 4 5 6 7 8 9" "Extremely full of energy" "Extremely without energy"

ASK2 "@humq06@At this moment, I am/feel:"

SCALE "1 2 3 4 5 6 7 8 9" "Extremely excited " "Extremely placid"

ASK2 "@humq07@At this moment, I am/feel:"

SCALE "1 2 3 4 5 6 7 8 9" "Extremely good" "Extremely bad"

ASK2 "@humq08@At this moment, I am/feel:"

GTS "@quest_end@"

PRINT "Important: make sure that the main screen of the ECG mobile is green. If it is yellow, please refer to the troubleshooting sheet."

PTOUT 600

PRINT "Thank you!"

:end

END

SE

# Sample size calculation

For subject i (between-person component) and moment j (within-person component), we define the following parameters with the E terms referring to the error terms, which are assumed to follow a normal distribution and have a standard deviation s. WTP_B_ refers to workplace telepressure at the between-person level and WTP_W_ refers to workplace telepressure at the within-person level. WTP_i_ = WTP_B_ + E_Bi_ with E_Bi_ ~ N(0, sWTP_B_); WTP_ij_ = WTP_i_ + E_wj_ with E_wj_ ~ N(0, sWTP_W_); WL_ij_ = betaWTP_WL * WTP_ij_ + EWL_ij_ with EWL_ij_ ~ N(0, sWL); PC_ij_ = betaWTP_PC * WTP_ij_ + EPC_ij_ with EPC_ij_ ~ N(0, sPC); Y_ij_ = betaWTP_Y * WTP_ij_ + betaWL_Y * WL_ij_ + betaPC_Y * PC_ij_ +EY_ij_ with EY_ij_ ~ N(0, sY). The betaWL_Y and betaPC_Y expressing the direct effects of WL and PC on Y, respectively are determined by their assumed mediated proportion (MP) as follows: betaWL_Y = (betaWTP_Y/betaWTP_WL) * (MP_WL_/(1- MP_WL_-MP_PC_)); betaPC_Y = (betaWTP_Y/betaWTP_PC) * (MP_PC_/(1-MP_WL_-MP_PC_)).

From Cambier and colleagues [1, 2], we know that sWTP_B_ and sWTP_W_ can be assumed to be equal. Thus, the power of the study corresponding to the presumed model depends on nine parameters: the four-variance parameters and the five regression coefficients (or equivalently, three regression coefficients and two percentages mediated). However, both the coefficients and the variances are to some extent arbitrary as they depend on the metric used and the unknown population variances. Moreover, multiplying every parameter by a constant (change of unit) would yield the same statistical power. Thus, in order to be able to have interpretable results, we considered the scenarios based on their interpretation in terms of the η^2^ effect sizes [3]. Note that η^2^ corresponds to the proportion of variance explained by an independent variable in a given regression model. It is equal to the R^2^ if only one independent variable is included. We varied the different parameters and generated thus 4212 combinations of the parameters for which we determined the different effect sizes. We set MP to be between 20% and 30% as we consider this to be scientifically meaningful. The effect size corresponding to the global univariate effect of WTP on Y was always the lowest of the different effect sizes. We first excluded all scenarios for which any of the effect was greater than 20%, thus selecting 1812 scenarios. We then selected among these the scenarios for which the effect size for the global univariate effect of WTP on Y was between 2% and 5% - 60 scenarios were thus selected. All effect sizes for these scenarios would be considered small according to Cohen J.’s [3] criteria for interpreting η^2^. Finally, by running 1000 simulations of the aforementioned model for the combinations of the parameters of the 60 selected scenarios with sample sizes between 100 and 150, we determined that these effects are significant at α = .05 and a power >.80 for all scenarios with a sample equal or greater than 120 participants.

We are therefore confident that a study with a sample size of 120 is sufficiently powered to detect any reasonably sized effect. In order to account for differences in WTP during weekends, WTP was assumed to stay at the same level for 10% of the subjects and to diminish by half of the weekly standard deviation of WTP for the other 90% of the subjects.

**References**

1. Cambier R, Derks D, Vlerick P: **Detachment from Work: A Diary Study on Telepressure, Smartphone Use and Empathy**. *Psychol Belg* 2019, **59**(1):227-245.

2. Cambier R, Vlerick P: **When Thoughts Have No Off Switch: The Cost of Telepressure and Message-based Communication behaviour within Boundary-crossing Contexts**. *Occup Health Sci* 2022, **6**(4):545-564.

3. Cohen J: **Statistical Power Analysis for the Behavioral Sciences**, 2nd ed. edn: Lawrence Erlbaum Associates; 1988.
